# RAGE activation in macrophages and development of experimental diabetic polyneuropathy

**DOI:** 10.1172/jci.insight.160555

**Published:** 2022-12-08

**Authors:** Sho Osonoi, Hiroki Mizukami, Yuki Takeuchi, Hikari Sugawa, Saori Ogasawara, Shizuka Takaku, Takanori Sasaki, Kazuhiro Kudoh, Koichi Ito, Kazunori Sango, Ryoji Nagai, Yasuhiko Yamamoto, Makoto Daimon, Hiroshi Yamamoto, Soroku Yagihashi

**Affiliations:** 1 Department of Pathology and Molecular Medicine and; 2 Department of Endocrinology and Metabolism, Hirosaki University Graduate School of Medicine, Hirosaki, Japan.; 3 Laboratory of Food and Regulation Biology, Department of Bioscience, School of Agriculture, Tokai University, Higashi-ku, Kumamoto, Japan.; 4 Diabetic Neuropathy Project, Department of Diseases and Infection, Tokyo Metropolitan Institute of Medical Science, Setagaya-ku, Tokyo, Japan.; 5 Department of Bioscience and Laboratory Medicine, Hirosaki University Graduate School of Health Sciences, Hirosaki, Japan.; 6 Department of Biochemistry and Molecular Vascular Biology, Kanazawa University Graduate School of Medical Sciences, Kanazawa, Japan.; 7 Komatsu University, Komatsu, Japan.

**Keywords:** Endocrinology, Neuroscience, Diabetes, Neurodegeneration, Neuroendocrine regulation

## Abstract

It is suggested that activation of receptor for advanced glycation end products (RAGE) induces proinflammatory response in diabetic nerve tissues. Macrophage infiltration is invoked in the pathogenesis of diabetic polyneuropathy (DPN), while the association between macrophage and RAGE activation and the downstream effects of macrophages remain to be fully clarified in DPN. This study explored the role of RAGE in the pathogenesis of DPN through the modified macrophages. Infiltrating proinflammatory macrophages impaired insulin sensitivity, atrophied the neurons in dorsal root ganglion, and slowed retrograde axonal transport (RAT) in the sciatic nerve of type 1 diabetic mice. RAGE-null mice showed an increase in the population of antiinflammatory macrophages, accompanied by intact insulin sensitivity, normalized ganglion cells, and RAT. BM transplantation from RAGE-null mice to diabetic mice protected the peripheral nerve deficits, suggesting that RAGE is a major determinant for the polarity of macrophages in DPN. In vitro coculture analyses revealed proinflammatory macrophage–elicited insulin resistance in the primary neuronal cells isolated from dorsal root ganglia. Applying time-lapse recording disclosed a direct impact of proinflammatory macrophage and insulin resistance on the RAT deficits in primary neuronal cultures. These results provide a potentially novel insight into the development of RAGE-related DPN.

## Graphical abstract

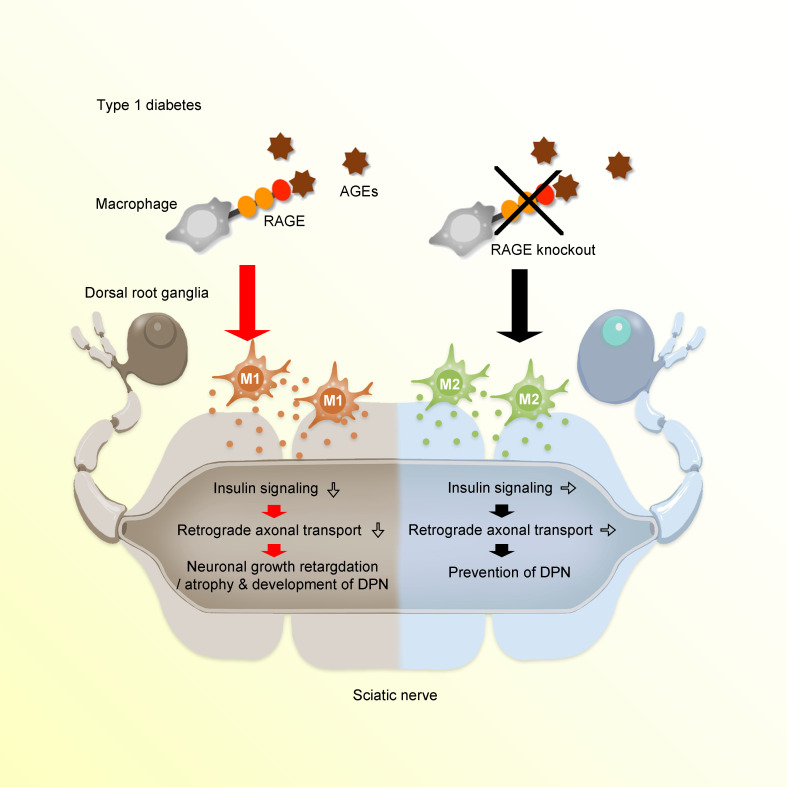

## Introduction

Diabetic polyneuropathy (DPN) is an early and prevalent complication of diabetes, affecting approximately 35%–50% of diabetic patients (1). DPN is a complex disorder in which the pathology develops through the dysregulation of multiple biochemical pathways secondary to chronic hyperglycemia. Activation of the polyol pathway and protein kinase C; increased oxidative stress and glycation; glucosamine accumulation; and low-grade inflammation are considered to be the main pathways involved, but an effective treatment based on the precise pathogenesis has yet to be established (2–5).

Advanced glycation end products (AGEs) are heterogeneous molecules derived from the reaction of glucose or other sugar derivatives with proteins or lipids that bind to receptors for AGEs (RAGE). Activation of RAGE has been commonly indicated as a major pathogenic factor in diabetic complications and neurodegenerative conditions (6–10). RAGE triggers an increase in cytokines, oxidative stressors, and proinflammatory molecules through macrophage activation, eliciting the polarization of macrophages into proinflammatory macrophage (M1) (9–12). General inflammation induced by M1 macrophage infiltration is a causative factor of diabetes, particularly type 2 diabetes (T2D) (13, 14). Such inflammatory conditions are associated with insulin resistance, particularly in the fatty tissue of T2D patients (14). Moreover, low-grade inflammation exerted by M1 characterizes the pathology of DPN (15–19). Although inflammation and the dysregulation of insulin signaling are thought to be mechanistic factors of DPN, few studies have explored their detailed associations with the pathophysiology of DPN in type 1 diabetes (T1D) (2, 20–23).

Retrograde axonal transport (RAT) is an essential function of neuronal maintenance and survival (24, 25). In experimental DPN, RAT is first impaired, and anterograde transport is subsequently reduced (26). On the other hand, impairment of RAT of nerve growth factor (NGF) has been reported, while RAT of stress kinases such as JNK and p38 is enhanced (27, 28). These results indicate that the stages of DPN or cargo-specific changes in RAT may be induced in DPN. Dysregulation of insulin signaling and concomitant inflammation are thought to impair RAT dynamics (25, 29–32). In DPN, cytological stress induced by hyperglycemia can cause RAT deficits, while the involvement of inflammation and insulin resistance remains putative (33).

In the present study, we aimed to clarify the role of macrophage-associated inflammation triggered by activation of the RAGE pathway in DPN and focused on changes in insulin signaling and RAT. The findings of this study shed light on the significance of RAGE activation in DPN pathology of T1D and suggest that controlling inflammation by modulating RAGE may be a novel therapeutic strategy.

## Results

### Diabetic RAGE-null mice are protected from the onset of DPN.

The mRNA of *Rage* was most abundantly expressed in the lung, but it was also expressed in the sciatic nerve (SN) and the dorsal root ganglia (DRG) (Supplemental Figure 1A; supplemental material available online with this article; https://doi.org/10.1172/jci.insight.160555DS1). *Rage* expression was blunted in RAGE-null mouse (RN) tissues compared with WT mouse tissues. (Supplemental Figure 1B). Body weight, fed blood glucose, nerve conduction velocities (NCVs), tail flick latency, intraepidermal nerve fiber density (IENFD), and nerve fiber area in the SN were comparable between WT and RN at baseline (Table 1, Supplemental Table 1, and Supplemental Figure 2, A–F). After 8 weeks, body weight, NCVs, axons, and myelin area were significantly increased in the nondiabetic groups. At 8 weeks of streptozotocin-induced (STZ-induced) diabetes, fed blood glucose levels were increased by approximately 32 mmol/L, while body weight was not increased in diabetic WT mice (WT-DM) or diabetic RAGE-null mice (RN-DM) compared with their nondiabetic counterparts. Increases in axon and myelin area were also not evident in WT-DM compared with WT mice and were maintained in RN-DM (*P* < 0.05 versus WT and WT-DM). Diabetes did not show impacts on small fiber function and structure in WT or RN. No impaired thermal responses in WT-DM were observed in the tail flick test. IENFD in the plantar skin was comparable between WT-DM and WT–non-DM (Table 1 and Supplemental Figure 3). Similarly, those assessments for small fibers in RN-DM did not show any impairments compared with non-DM littermates. Serum AGEs were evaluated by electrospray ionization liquid chromatography–tandem mass spectrometry (LC-MS/MS) (34). Mice in both diabetic groups showed 1.5-fold higher serum Nε-carboxymethyl lysine (CML) concentrations than their nondiabetic counterparts.

### Infiltration of macrophages and the modulation of macrophage polarity in the SN in DPN.

**Figure 1:**
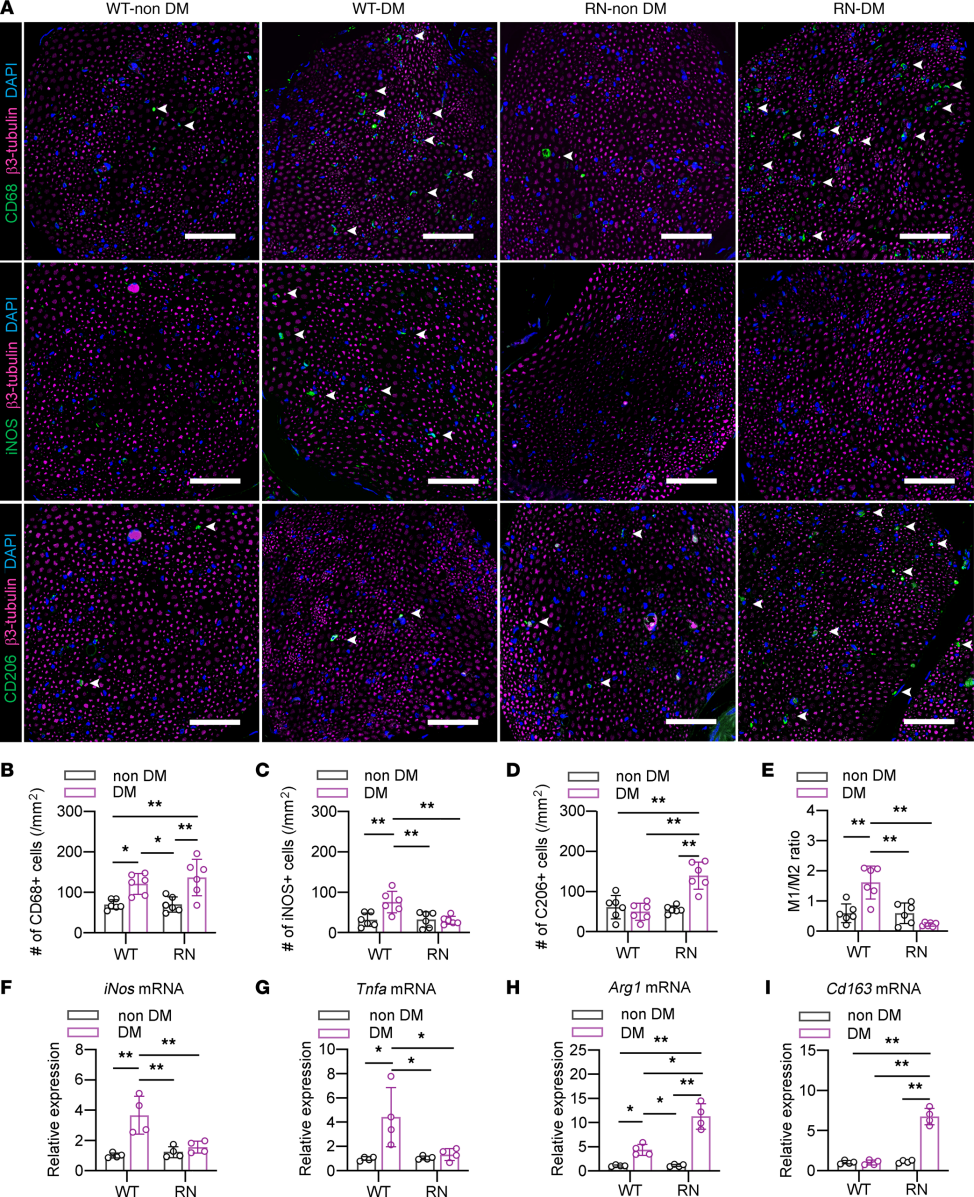
The polarity of infiltrating macrophages is altered by diabetes and RAGE signalling in the sciatic nerve. (**A**) Representative images of the sural nerve (SN) of mice from each group at 8 weeks after diabetes induction that were immunostained for β3-tubulin and CD68 (upper), iNOS (middle), or CD206 (lower). Scale bar: 50 μm. Arrowheads indicate infiltrated macrophages. (**B**–**E**) Quantitative analyses of SN infiltration of CD68^+^ macrophages (**B**), iNOS^+^ M1 macrophages (**C**), CD206^+^ M2 macrophages (**D**), and the ratio of M1/M2 macrophages (**E**). (**F**–**I**) Relative mRNA expression of activated M1 and M2 macrophage markers in the SN. *iNos* (**F**) and *Tnfa* (**G**) indicate M1 macrophages, and *Arg1* (**H**) and *Cd163* (**I**) indicate M2 macrophages. The data are presented as the mean ± SD; *n* = 6 mice/group for immunostaining and *n* = 4 mice/group for qPCR. Statistical analysis was performed by 2-way ANOVA with post hoc multiple-comparison tests. *P < 0.05, **P < 0.01.

Eight weeks after diabetes induction, the number of cells positive for the panmacrophage marker CD68 was increased in the SN of diabetic mice compared with that of their nondiabetic counterparts, despite the difference in genotypes (Figure 1, A and B). Regarding M1 macrophage infiltration, the number of inducible nitric oxide synthase–positive (iNOS^+^) cells and the mRNA expression of *iNos* and *Tnfa* were significantly increased in the SN of WT-DM compared with WT–non-DM and RN-DM, while the number and expression levels were comparable between RN and RN-DM (Figure 1, A, C, F, and G). The number of iNOS^+^ macrophages was negatively correlated with NCVs, indicating that the activation of RAGE signaling might be associated with the severity of DPN (Supplemental Figure 4). Conversely, the number of CD206^+^ antiinflammatory macrophages (M2) and the mRNA expression of *Arg1* and *Cd163* were significantly increased in the SN of RN-DM compared with RN–non-DM and WT-DM (Figure 1, A, D, H, and I). Furthermore, the M1/M2 ratio was calculated based on the number of macrophages and was significantly increased in the SN of WT-DM, while it was comparable in the SN of WT–non-DM, RN–non-DM, and RN-DM (Figure 1E).

### Activation of the inflammatory pathway and insulin signaling dysregulation in DPN.

**Figure 2:**
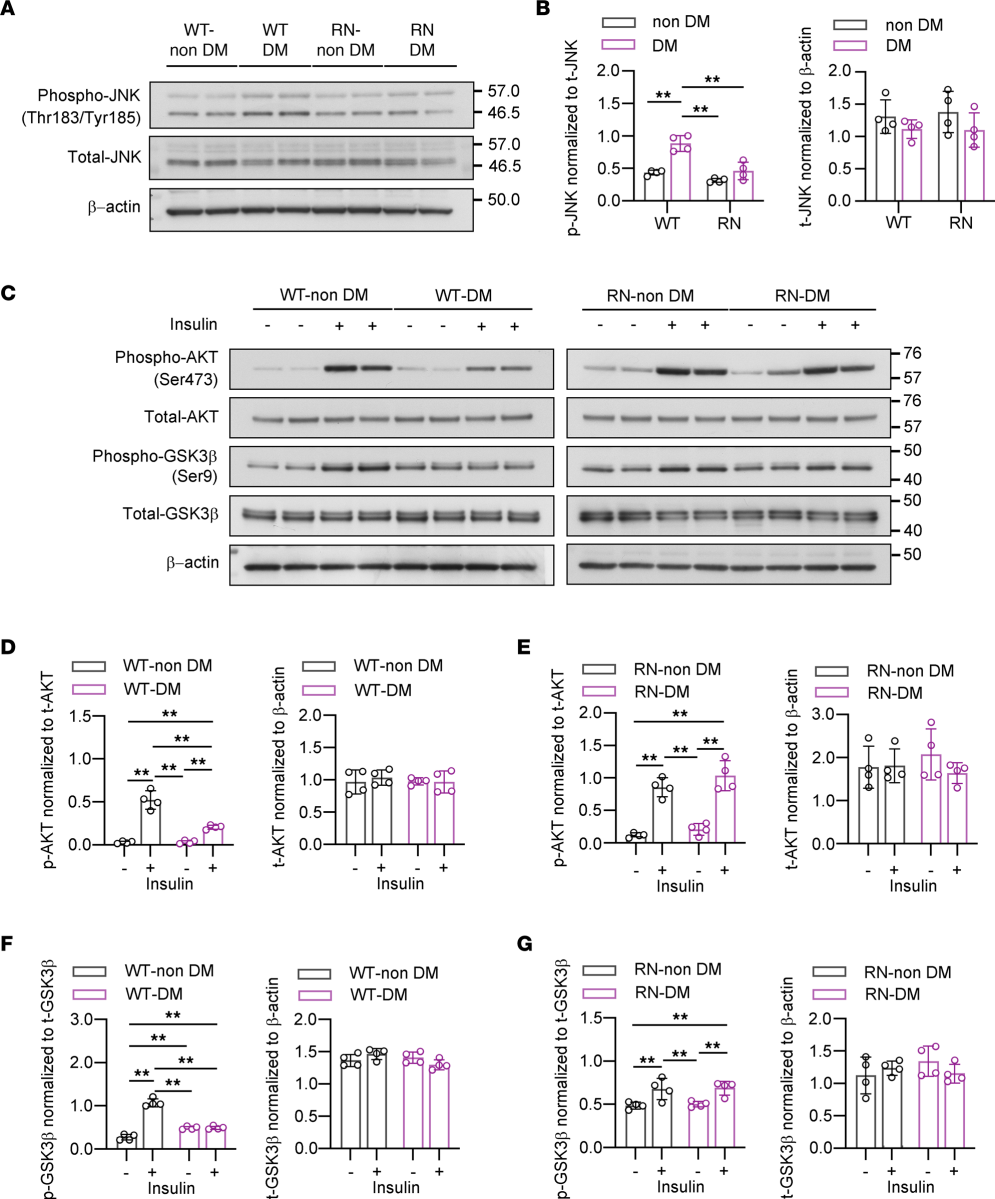
Elevated JNK phosphorylation and dysregulated insulin signaling are inflammatory features in the sciatic nerve in diabetic polyneuropathy, but RAGE depletion restores these abnormal changes. (**A**) Immunoblots showing JNK phosphorylation in the sural nerve (SN). (**B**) Quantification of phospho-JNK and total JNK in the immunoblots. (**C**) Immunoblots showing the phosphorylation of AKT and GSK3β in the SN. (**D**–**G**) Quantification of phospho-AKT and total AKT (**D** and **E**) and phospho-GSK3β and total GSK3β (**F** and **G**) in the immunoblots. The data are presented as the mean ± SD; *n* = 4 mice/group. Statistical analysis was performed by 2-way ANOVA with post hoc multiple-comparison tests. **P < 0.01.

M1-induced inflammation and subsequent upregulation of the TNF-α/JNK pathway disrupt insulin signaling, causing insulin resistance (IR) in diabetic adipose tissue (35, 36). We hypothesized that a similar mechanism might be involved in DPN pathology in T1D. To further strengthen our premise, we investigated the phosphorylation of JNK and molecules related to insulin signaling by Western blotting. After 8 weeks of diabetes, there was an increase in the expression of phosphorylated JNK in WT but not in RN, suggesting that the increased M1 macrophage infiltration might trigger local inflammation in the SN of WT-DM (Figure 2, A and B). We then investigated the insulin/AKT/GSK3β pathway, which is a central insulin signaling pathway that is attenuated in insulin resistance (37). The SN of WT-DM showed blunted phosphorylation of AKT in response to i.v. stimulation with 5U insulin for 10 minutes compared with that of WT–non-DM, while those of RN–non-DM and RN-DM were comparable (Figure 2, C–E). Although the SN of WT-DM showed increased GSK3β phosphorylation at the basal state, phosphorylation was decreased in response to insulin stimulation when compared with WT–non-DM. On the other hand, it was equivalent in the SN of RN–non-DM and RN-DM, as well as in the noninsulin-stimulated state (Figure 2, C, F and G).

### Deficits in RAT in the SN manifest in DPN.

**Figure 3:**
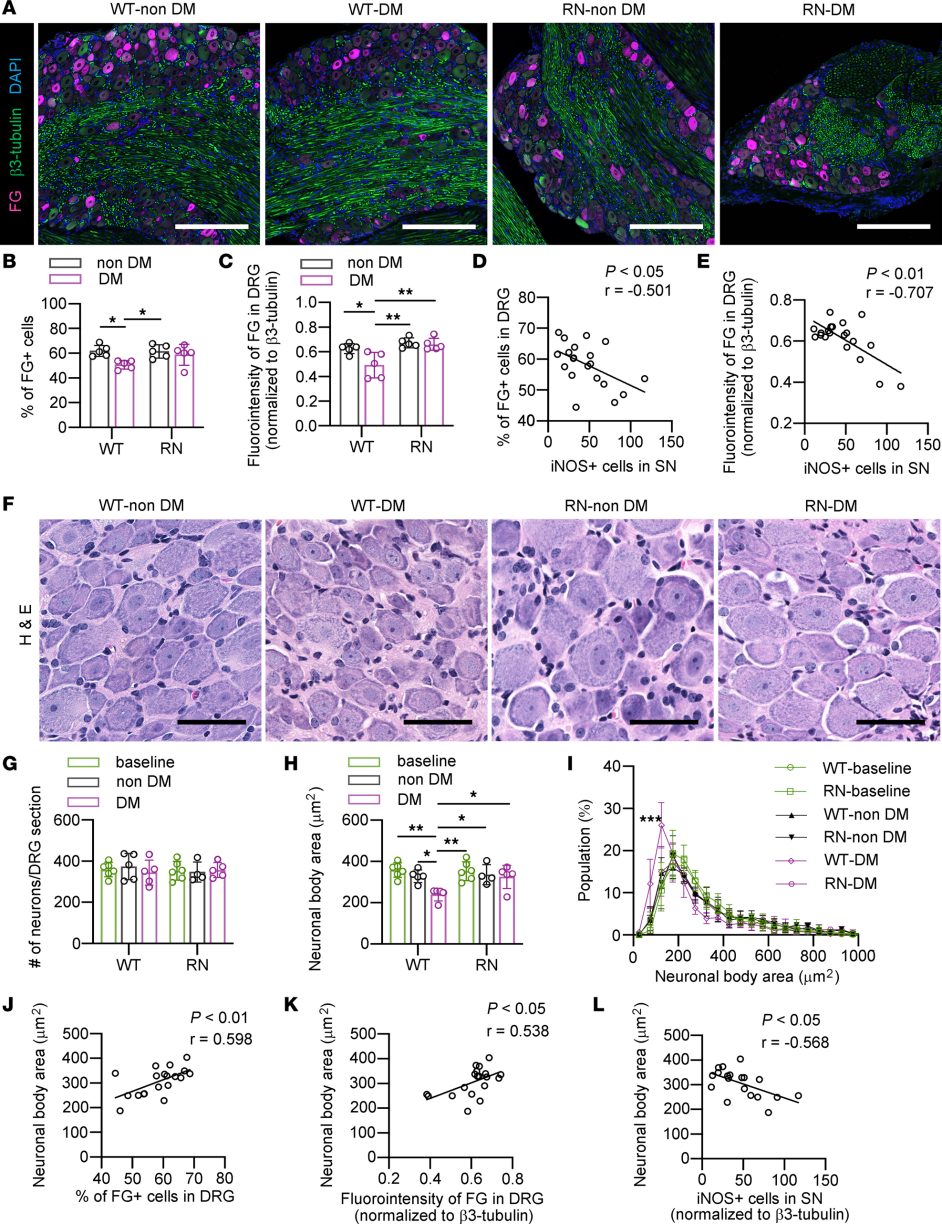
Impaired RAT and neuronal atrophy in dorsal root ganglia are restored in diabetic RAGE-null mice. (**A**) The dorsal root ganglia (DRG) immunostained for β3-tubulin and Fluoro Gold (FG) 5 days after FG injection. Scale bar: 200 μm. (**B** and **C**) The percentage of FG^+^ neurons, and the relative intensity of FG normalized to β3-tubulin in the DRG. (**D**) The correlation between the percentage of FG^+^ neurons and the number of iNOS^+^ cells in the sural nerve (SN). (**E**) The correlation between the relative intensity of FG and the number of iNOS^+^ cells in the SN. (**F**) Representative H&E staining of the DRG of mice after 8 weeks of diabetes. Scale bar: 50 μm. (**G**) Neuronal cell number in the DRG. (**H**) Average neuronal body area. (**I**) The histogram of neuronal body area in the DRG. The *x* axis shows the neuronal size distribution divided by every 50 μm^2^. The *y* axis shows the percentage of neurons for each size. (**J**) The correlation between the percentage of FG^+^ neurons and average neuronal body area. (**K**) The correlation between the relative intensity of FG and average neuronal body area. (**L**) The correlation between the average neuronal body area and the number of iNOS^+^ cells in the SN. The data are presented as the mean ± SD; *n* = 5 mice/group for **B** and **C**, *n* = 4–5 mice/group for **G** and **H**. Statistical analysis was performed by 2-way ANOVA with post hoc multiple-comparison tests for **B**, **C**, and **G**–**I** and by Pearson’s correlation analysis for **D**, **E**, and **J**–**L**. *P < 0.05, **P < 0.01, ***P < 0.001 versus other group than WT-DM.

Various protein kinases have been implicated in the regulation of axonal transport through phosphorylation of motor proteins, including kinesin and dynein, and components of motor protein complexes (38). TNF-α stimulates JNK, which directly phosphorylates motor proteins and impairs anterograde axonal transport and RAT (29, 39, 40). On the other hand, insulin signaling activates RAT through the phosphorylation of GSK3β and subsequent promotion of dynein motility (31, 41). Despite these findings, there is not yet a unified view of the changes in RAT in DPN (27, 28). After the retrograde nerve tracer Fluoro-Gold (FG) was injected into a hindlimb footpad in vivo (42, 43)*,* the profiles of RAT to the DRG through the SN were analyzed in DRG sections that were immunostained with an anti-FG antibody (Figure 3A). The number of FG^+^ neurons and the intensity of FG staining in the DRG were decreased in WT-DM compared with WT–non-DM. On the other hand, the profile of RAT in RN-DM was comparable with that of RN–non-DM (Figure 3, A–C). These findings indicated that RAT is maintained in RN-DM. The number of iNOS^+^ M1 macrophages in the SN and both RAT profiles showed an inverse correlation, suggesting the involvement of macrophage-induced inflammation in RAT deficits (Figure 3, D and E). Neuronal cell miniaturization in the DRG is another characteristic of DPN pathology and can be dependent on neurotrophic factors transported by RAT (23, 44). In contrast to the SN, morphological analysis showed that there was no significant increase in the number or average area of neuronal bodies in DRG neurons between 8 and 16 weeks of age for either WT or RN (Figure 3, F–I). Eight weeks of diabetes did not impact the number of neuronal cells, while the average area of the neuronal body was decreased with a left-shifted histogram based on the population of neuronal body area in the DRG of WT-DM compared with WT–non-DM, while RN-DM showed a similar average size and histogram of the neuronal body area. Both the number of FG^+^ neurons and the intensity of FG staining in the DRG were proportionally correlated with neuronal body area (Figure 3, J and K). The number of iNOS^+^ M1 macrophages in the SN was negatively correlated with neuronal body area in DRG (Figure 3L).

### Transplantation of RAGE-null BM partially suppresses the onset of DPN.

To further examine the specific role of RAGE in macrophages in DPN, we subjected WT mice to lethal irradiation and i.v. injected them with WT BM (BMWT) or RAGE-null BM (BMRN) (Supplemental Figure 5A). Reconstitution of the BM genotype was confirmed in the peripheral blood 8 weeks after transplantation (Supplemental Figure 5B), and diabetes was subsequently induced in the mice by STZ injection. Irradiation and transplantation of RAGE-null BM reduced the mRNA expression of *Rage* in the BM of BMRN–non-DM by 70%–80% (Supplemental Figure 5C). After 8 weeks of diabetes, BMWT-DM and BMRN-DM showed equivalent increases in blood glucose and body weight loss (Table 2). BMWT-DM showed significant delays in both motor and sensory NCVs, but no impaired thermal responses in the tail flick test and no significant reductions in IENFD were observed compared with those of BMWT–non-DM (Table 2 and Supplemental Figure 5D). Although BMRN-DM also showed significant delays in motor NCVs and a tendency of delay in sensory NCVs compared with those of BMRN–non-DM, both motor nerve conduction velocity (MNCV) and sensory nerve conduction velocity (SNCV) were significantly higher in RN-BMT than WT-BMT in diabetes. Other characteristics of DPN, including thermal response and IENFD, were maintained at levels equivalent to those of BMRN–non-DM.

### Diabetic mice transplanted with RAGE-null BM exhibit macrophage infiltration similar to that of RN-DM.

**Figure 4:**
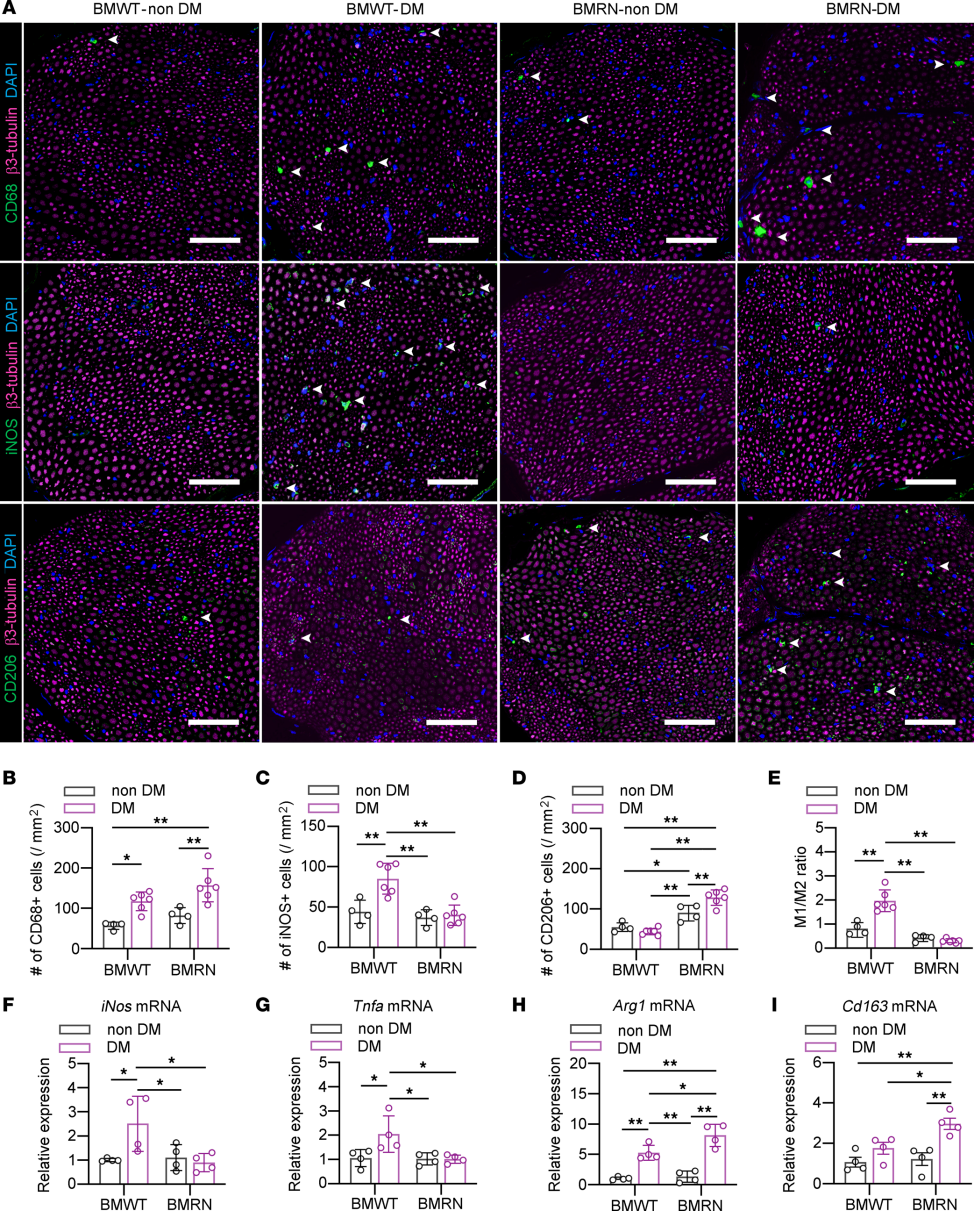
BM-specific depletion of RAGE alters the polarity of infiltrating macrophages in the sciatic nerve under diabetic conditions, mimicking whole-body depletion of RAGE. (**A**) Representative images of the sural nerve (SN) of mice from each group after 8 weeks of diabetes; the tissues were immunostained for β3-tubulin and CD68 (upper), iNOS (middle), or CD206 (lower). Scale bar: 50 μm. (**B**–**E**) Quantitative analyses of SN infiltration of CD68^+^ macrophages (**B**), iNOS^+^ M1 macrophages (**C**), CD206^+^ M2 macrophages (**D**), and the ratio of M1/M2 macrophages (**E**). (**F**–**I**) Relative mRNA expression of activated M1 and M2 macrophage markers in the SN. *iNos* (**F**) and *Tnfa* (**G**) indicate M1 macrophages, and *Arg1* (**H**) and *Cd163* (**I**) indicate M2 macrophages. The data are presented as the mean ± SD; *n* = 6 mice/group for **B**–**E**, *n* = 4 mice/group for **F**–**I**. Statistical analysis was performed by 2-way ANOVA with post hoc multiple-comparison tests. *P < 0.05, **P < 0.01.

CD68 immunostaining showed that the number of infiltrating macrophages was increased in the SN of diabetic BM-transplanted mice compared with that of their nondiabetic counterparts, regardless of genotype (Figure 4, A and B). The number of iNOS^+^ M1 macrophages and the mRNA expression of *iNos* and *Tnfa* were higher in the SN of BMWT-DM than in that of BMWT–non-DM (Figure 4, A, C, F, and G). The number of iNOS^+^ M1 macrophages was negatively correlated with NCVs (Supplemental Figure 6). On the other hand, the SN of BMRN-DM exhibited an increased number of CD206^+^ M2 macrophages and increased mRNA expression of *Arg1* and *Cd163* (Figure 4, A, D, H, and I). The M1/M2 ratio was significantly increased in the SN of BMWT-DM compared with that of the other groups (Figure 4E).

### Insulin signaling is maintained in the SN of WT mice transplanted with RAGE-null BM.

**Figure 5:**
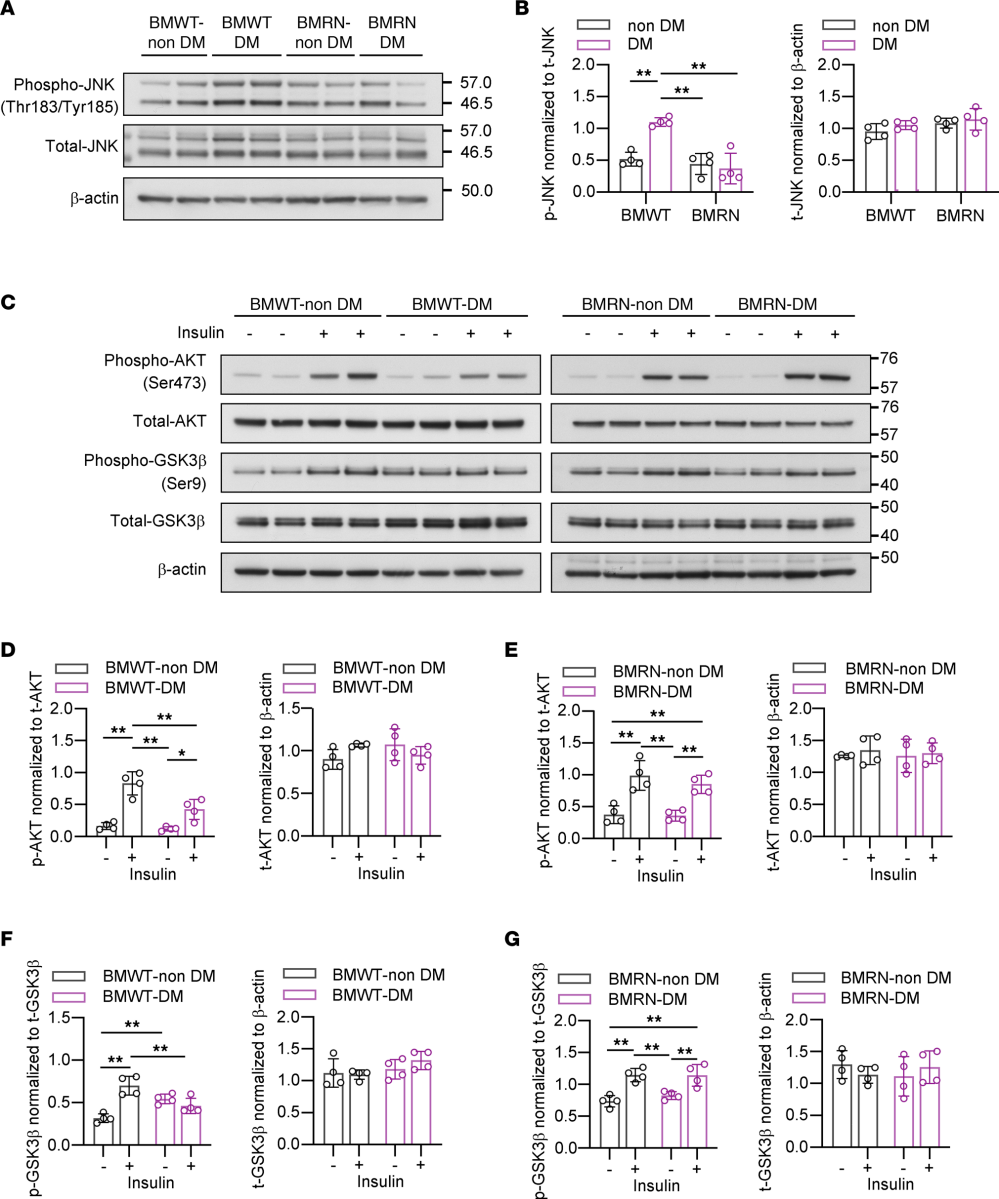
BM-specific depletion of RAGE restores inflammatory changes and dysregulated insulin signaling in the sciatic nerve in diabetic polyneuropathy. (**A**) Immunoblots showing JNK phosphorylation in the sural nerve (SN). (**B**) Quantification of phospho-JNK and total JNK in the immunoblots. (**C**) Immunoblots showing the phosphorylation of AKT and GSK3β in the SN. (**D**–**G**) Quantification of phospho-AKT and total AKT (**D** and **E**) and phospho-GSK3β and total GSK3β (**F** and **G**) in the immunoblots. The data are presented as the mean ± SD; *n* = 4 mice/group. Statistical analysis was performed by 2-way ANOVA with post hoc multiple-comparison tests. *P < 0.05, **P < 0.01.

Western blotting confirmed the increased phosphorylation of JNK in the SN of BMWT-DM compared with that in the remaining groups (Figure 5, A and B). Impairment of the insulin/AKT/GSK3β pathway was also confirmed in the SN of BMWT-DM (Figure 5, C, D, and F). In contrast, BM-specific KO of RAGE restored the abnormal phosphorylation of JNK and maintained the insulin/AKT/GSK3β pathway, similar to RN (Figure 5, C, E, and G).

### RAGE-null BM transplantation ameliorates the deficits in RAT in the diabetic SN.

**Figure 6:**
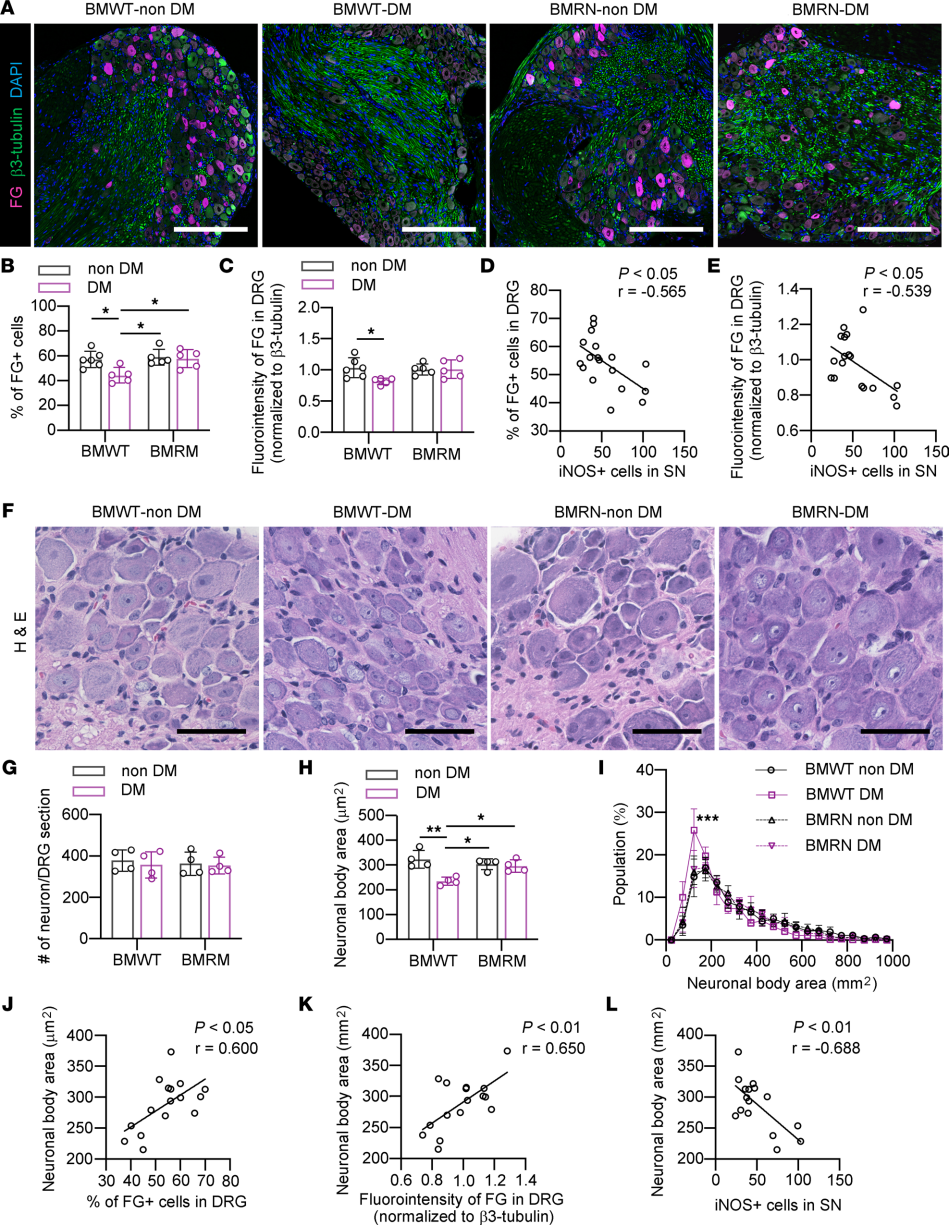
Impaired RAT and neuronal atrophy in dorsal root ganglia are restored in diabetic WT mice transplanted with RAGE-null mouse BM. (**A**) The dorsal root ganglia (DRG) immunostained for β3-tubulin and Fluoro Gold (FG) 5 days after FG injection after 8 weeks of diabetes. Scale bar: 200 μm. (**B** and **C**) The percentage of FG-positive neurons, and the relative intensity of FG in DRG. (**D**) The correlation between the percentage of FG^+^ neurons and the number of iNOS^+^ cells in the sural nerve (SN). (**E**) The correlation between the relative intensity of FG and the number of iNOS^+^ cells in SN. (**F**) Representative H&E staining of the DRG of mice after 8 weeks of diabetes. Scale bar: 50 μm. (**G** and **H**) Average neuronal number and body area in the maximum cross-section of DRG. (**I**) The histogram of neuronal body area. The *x* axis shows the neuronal size distribution divided by every 50 μm^2^. The *y* axis shows the percentage of neurons of each size. (**J** and **K**) The correlation between the percentage of FG^+^ neurons or the relative intensity of FG and average neuronal body area. (**L**) The correlation between the number of infiltrated iNOS^+^ cells in SN and average neuronal body area. The data are presented as the mean ± SD; *n* = 5 mice/group for **B** and **C**, *n* = 4–5 mice/group for **G**–**I**. Statistical analysis was performed by 2-way ANOVA with post hoc multiple-comparison tests for **B**, **C**, **G**, and **H** and by Pearson’s correlation analysis for **D**, **E**, and **J**–**L**. *P < 0.05, **P < 0.01, ***P < 0.001 versus other group than BMWT DM.

RAT was analyzed by FG labeling of DRG neurons, and the number of FG^+^ cells and the intensity of FG staining were significantly decreased in the DRG of BMWT-DM compared with BMWT–non-DM, while these levels were maintained in the DRG of BMRN-DM, which were comparable with those of BMRN–non-DM (Figure 6, A–C). Both RAT parameters were inversely correlated with the number of infiltrating iNOS^+^ M1 macrophages in the SN (Figure 6, D and E). Morphological analysis of the DRG showed no changes in neuronal cell numbers in DRG or neuronal cell atrophy in BMWT-DM, and this induced a left-side shift in the histogram for the neuronal body area. This was similarly observed in WT-DM, while the average size and histogram of shape for the neuronal body in BMRN-DM were comparable with those of BMRN–non-DM (Figure 6, F–I). Both the number of FG^+^ neurons and the intensity of FG staining in the DRG were proportionally correlated with neuronal body size (Figure 6, J and K). The number of iNOS^+^ M1 macrophages in the SN was negatively correlated with neuronal body area in the DRG (Figure 6L).

### Activated proinflammatory macrophages impair RAT.

**Figure 7:**
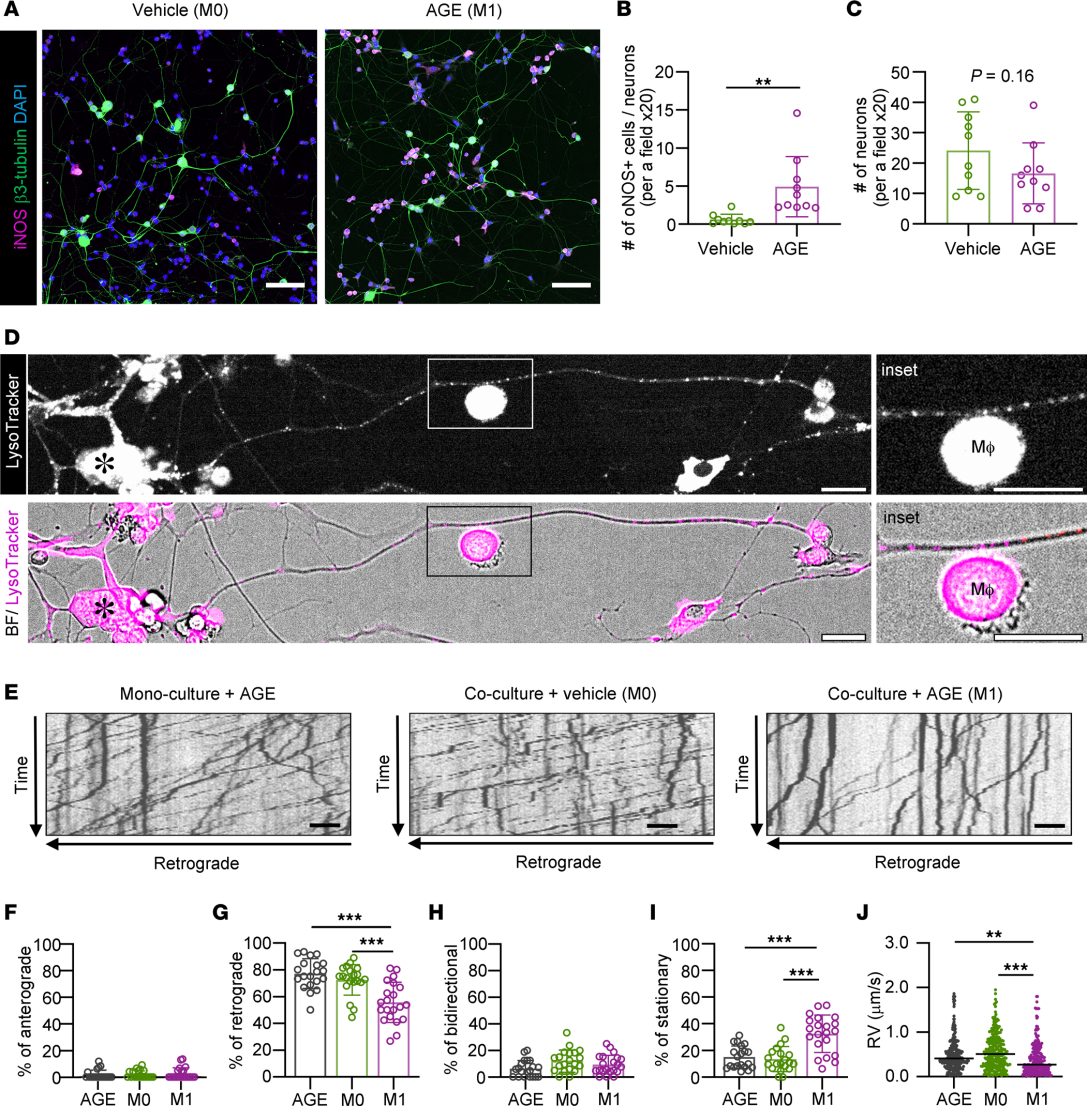
AGEs induce macrophage polarization toward a proinflammatory phenotype. Macrophage-related inflammation impairs RAT in DRG neurons. (**A**) Immunofluorescent images showing the expression of iNOS in RAW264.7 cells. Neurons were immunostained for β3-tubulin. Scale bar: 100 μm. (**B**) The number of iNOS^+^ cells (M1) normalized to that of dorsal root ganglia (DRG) neurons per field. *n* = 10 fields (×20)/group from 3 independent experiments. (**C**) The number of DRG neurons immunostained for β3-tubulin per field after treatment with vehicle or AGEs for 12 hours. *n* = 10 fields (×20)/group from 3 independent experiments. (**D**) A representative image of cocultures of DRG neurons and RAW264.7 cells labeled with LysoTracker Red. Images of DRG neurons were captured at 2-second intervals for 4 minutes. Scale bar: 50 μm. (**E**) Kymographs of LysoTracker-labeled organelles in axons from DRG neuron monocultures treated with AGEs for 12 hours or from DRG neuron-RAW264.7 cell cocultures treated with vehicle or AGEs for 12 hours. Scale bar: 10 μm. The horizontal arrow indicates the retrograde direction for the 100 μm axon segment. The vertical arrow indicates total recording time (4 minutes). (**F**–**I**) The percentage of organelles in 100 μm axon segments that moved anterogradely (**F**), retrogradely (**G**), bidirectionally (**H**), or were stationary (**I**) in each treatment condition. *n* = 18–21 axons from 3 independent experiments. (**J**) The velocity of retrograde movement in 100 μm axon segments in each treatment condition. The data consisted of 200–300 movements. The data are presented as the mean ± SD. Statistical analysis was performed by Student’s unpaired 2-tailed *t* test for **B** and **C** and by 1-way ANOVA with Tukey’s multiple-comparison test for **F**–**J**. *P < 0.05, **P < 0.01, ***P < 0.001.

The increased mRNA expression of proinflammatory molecules and the effects of M1-induced inflammation may be involved in axonal transport deficits in DPN. To verify the direct effects, we used a coculture system in which primary sensory neurons from the DRG of WT and the macrophage cell line RAW264.7 directly contacted each other (Figure 7A and Supplemental Figure 7A). RAW264.7 cells were activated and polarized into M1-expressing iNOS and TNF-α cells by LPS and AGE, and the effect of AGE was less than that of LPS (Supplemental Figure 7, B and C). RAW264.7 cells activated by AGE showed morphological changes, such as spindle or ameboid shapes, in the coculture system compared with vehicle stimulation (Supplemental Figure 7D). In contrast, high mobility group box-1 protein (HMGB1) failed to activate macrophages. When treated with AGE, immunofluorescence revealed that the number of RAW264.7 cells expressing iNOS was increased in the coculture system (Figure 7, A and B), but the number of neurons was not altered (Figure 7, A and C). To evaluate changes in axonal transport, intraaxonal vesicles were labeled using LysoTracker Red, and their motility was observed by time-lapse microscopy (Figure 7D). LysoTracker Red labels acidic organelles, including lysosomes, late endosomes, and autophagosomes, which are predominantly transported in a retrograde manner (45, 46). The movies obtained from time-lapse images acquired every 2.0 seconds for 4 minutes (121 frames) were analyzed, and kymographs were generated from a 100 μm segment of the mid-axon (Figure 7E). The slope, length, and direction of each line provide information about the velocity, run length, and direction of the movement of individual vesicles. The movement of vesicles was classified into 4 categories: anterograde (≥5 μm; lines sloped and to the right), retrograde (≥5 μm; lines sloped to the left), bidirectional (zigzagged lines), and stationary (<5 μm). In the monoculture of DRG neurons, AGE treatment did not affect the direction or average velocity of retrograde movement (Figure 7, F–J, and Supplemental Video 1). Approximately 70% of vesicles showed retrograde movement in the vehicle-treated culture (M0) (Supplemental Video 2). On the other hand, AGE treatment (M1) reduced the percentage and average velocity of retrograde movement and increased the number of stationary vesicles in the coculture (Figure 7, F–J, and Supplemental Video 3).

### Activation of the TNF-α/JNK pathway impairs insulin signaling and RAT.

**Figure 8:**
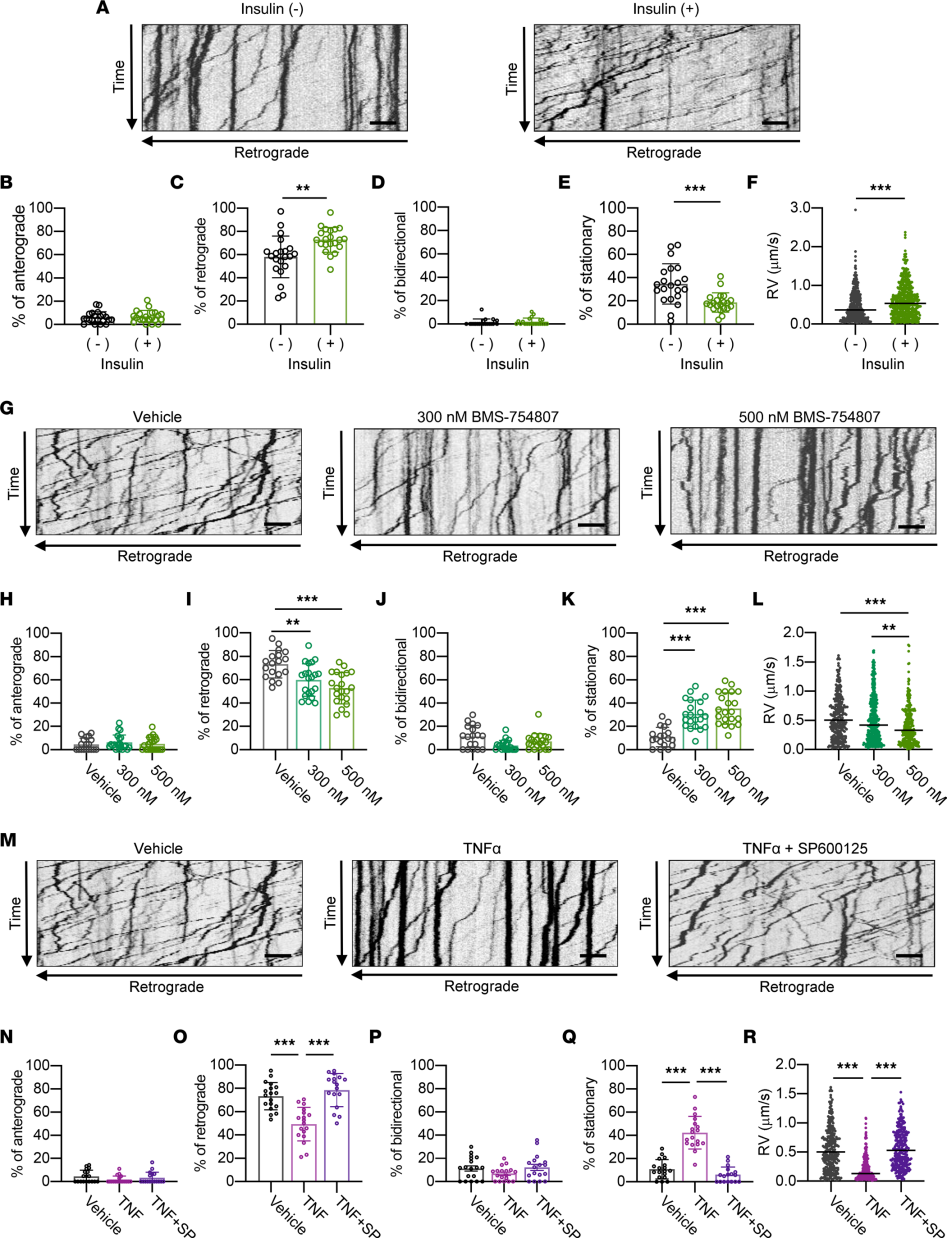
Disruption of insulin signaling impairs RAT in DRG neurons. (**A**) Kymographs of LysoTracker-labeled organelles in axons from dorsal root ganglia (DRG) neurons with 1.0 U/mL insulin. The horizontal and vertical arrows indicate retrograde direction and recording time (4 minutes), respectively. (**B**–**E**) The percentage of organelles in 100 μm axon segments that moved anterogradely (**B**), retrogradely (**C**), bidirectionally (**D**), or were stationary (**E**). *n* = 18–21 axons from 3 independent experiments. (**F**) The velocity of retrograde movements (RV) in 100 μm axon segments. The data consisted of 200–300 movements. (**G**) Kymographs in axons from DRG neurons treated with vehicle and insulin receptor antagonist (BMS-754807, 300 or 500 nmol/L). The stimulation time was 60 minutes. The horizontal and vertical arrows indicate retrograde direction and recording time (4 minutes), respectively. (**H**–**K**) The percentage of organelles in 100 μm axon segments that moved anterogradely (**H**), retrogradely (**I**), or bidirectionally (**J**), or were stationary (**K**). *n* = 18–21 axons from 3 independent experiments. (**L**) RV in 100 μm axon segments in each treatment condition. (**M**) Kymographs in axons from DRG neurons treated with vehicle, TNF-α, and TNF-α + JNK inhibitor (SP600125). The stimulation time was 20 minutes. The vertical arrow indicates recording time (4 minutes). (**N**–**Q**) The percentage of organelles in 100 μm axon segments that moved anterogradely (**N**), retrogradely (**O**), bidirectionally (**P**), or were stationary (**Q**). *n* = 18–21 axons from 3 independent experiments. (**R**) RV in 100 μm axon segments. The data consisted of 200–300 movements. The data are presented as the mean ± SD. Because the experiments of **G**–**L** and **M**–**R** were performed contemporaneously, statistical analysis was done using same vehicle control. Statistical analysis was performed by Student’s 2-tailed unpaired *t* test for **B**–**F** and by 1-way ANOVA with Tukey’s multiple-comparison test for **H**–**L** and **N**–**R**. **P < 0.01, ***P < 0.001.

To further explore the effects of humoral factors secreted by M1 macrophages on RAT, we investigated the effects of TNF-α on the molecular changes of insulin signaling elicited by TNF-α in DRG neuron monocultures. Alterations in protein kinase activity in axons labeled with β3-tubulin were investigated by immunofluorescence. TNF-α stimulation upregulated the phosphorylation of JNK in the axons of DRG neurons (Supplemental Figure 8, A and B). Insulin/AKT/GSK3β signaling was also evaluated in this model. TNF-α stimulation downregulated the insulin-induced phosphorylation of AKT and GSK3β in the axons of DRG neurons (Supplemental Figure 8, C–F). Next, we explored whether insulin signaling directly regulates axonal transport. The depletion of insulin from the culture media reduced the percentage and average velocity of retrograde movement and increased stationary vesicles in monocultures (Figure 8, A–F, and Supplemental Videos 4 and 5). Similarly, antagonization of insulin and IGF-I receptors with BMS-754807 reproduced the alterations in axonal transport under insulin-null conditions (Figure 8, G–L, and Supplemental Videos 6, 9, and 10). Finally, axonal transport in response to TNF-α was evaluated. TNF-α stimulation also reduced the percentage and average velocity of retrograde movement and increased stationary vesicles in monocultures, and this effect was abrogated by treatment with both TNF-α and SP600125, a JNK inhibitor (Figure 8, M–R, and Supplemental Videos 7 and 8). These results support our hypothesis that M1-associated humoral factors also impair insulin signaling in the peripheral nervous system, leading to neuronal dysfunction, including RAT deficits (Supplemental Figure 9, A–C).

## Discussion

Previous studies have reported that RN with a short duration of diabetes (2–3 months) are protected from delay of NCVs, while those with a long duration of diabetes (6 months) are partially protected from the loss of perception and SNCV but not PGP 9.5^+^ nerve fiber loss, according to suppression of NF-κB activation in DRG neurons (47–49). In this study, we reported that RAGE deletion in the BM suppressed the development of DPN in a STZ-induced T1D model in short duration (8 weeks), suggesting a role for macrophages in their genesis.

The effectiveness of low-dose insulin treatment has been established in T1D experimental DPN models without the normalization of blood glucose levels (48, 50). Decreased insulin or dysregulation of insulin signaling could be therapeutic targets in DPN in T1D, because insulin resistance is characteristic in the SN and DRG in both T1D and T2D (21, 23). Our previous study revealed that the activation of plasma xanthine oxidase induces M1 macrophage polarization and exacerbates DPN in *db/db* mice (19). Our results first showed that M1 macrophage polarization via RAGE induced insulin resistance in the peripheral nervous system in the early stage of T1D DPN. Nevertheless, intranasal insulin administration from 8 to 16 weeks of diabetes provided no additional effects on DPN compared with simple RAGE deletion in STZ-induced diabetic RN mice (48). Taking into account the degrees of macrophage infiltration (maximum approximately 8 weeks of diabetes then dropping), the effects of RAGE inhibition on insulin sensitivity may be attenuated after 8 weeks of diabetes. This may indicate that the timing of insulin administration is important for most RAGE signals in the early stage of DPN treatment.

Insulin has shown trophic effects on DRG neurons in many in vitro and in vivo studies (22, 23, 48, 50). Conversely, Grote et al. reported that the effects of physiological insulin on the development and function of nerve tissue are limited to insulin receptor–null mice and are specific for sensory nerves (51). These findings indicate that defects in insulin signaling in peripheral nerves can be compensated with insulin-like growth factor 1 (IGF-1) because IGF-1 receptors and insulin receptors can form heterodimers and have common signaling factors. Hackett et al. showed that the concurrent deletion of insulin and IGF-1 receptors in Schwann cells induces myelin thinning and delays NCVs (52). Because the inhibition of insulin and IGF-1 receptor with BMS-754807 dose-dependently attenuated the RAT of acidic organelles, the RAT of acidic organelles can be regulated coordinately by insulin and IGF-1 signaling. Considering that insulin and IGF-1 resistance are generally observed in the diabetic state, M1 macrophages activated by RAGE may induce both insulin and IGF-1 resistance in peripheral nerve tissues.

WT mice showed a progressive increase in body weight, NCVs, and nerve fiber area up to 16 weeks of age, while diabetic mice exhibited a smaller increase, resulting in significant differences at the end of the experiments. These findings suggest that maturity disorder contributes to the deficit of nerve function, as shown in previous reports (53, 54). In contrast, the average area of neuronal body in the DRG was comparable between 8 and 16 weeks of age, as reported previously (55), while those in WT-DM were significantly decreased without changing the number of neuronal cells at the end of the experiments. These results implied that atrophy, rather than growth retardation or cell death, could be involved in the miniaturization and dysfunction of neuronal cells in our study. Thus, growth retardation and atrophy may be involved in the deficit of nerve function in our model. Because insulin is a neurotrophic factor, reactivation of insulin signaling achieved by the deletion of RAGE can promote local nerve growth, as well as prevent of atrophy of neuronal cells via RAT for neurotrophic molecules.

Axonal transport is an essential function for neuronal maintenance and survival in the peripheral nervous system and is regulated by an elaborate mechanism associated with the direction, velocity of transport, and cargo class (24). Deficits in axonal transport — such as slow anterograde axonal transport and RAT and mitochondrial motility — have been described in the context of DPN pathology, while precise mechanisms are still largely unknown (9, 25–27, 33). In RAT, the dynein complex and protein kinases, including JNK, ERK, AKT, GSK3β, and CDK5, are involved in regulating movement (38). In this study, direct stimulation with TNF-α phosphorylated JNK, resulting in RAT impairment in vitro, as shown in previous reports (29, 39, 40). On the other hand, TNF-α stimulation attenuated insulin signaling in neuronal cells. GSK3β is located downstream of insulin and JNK signaling, which phosphorylates components of the dynein complex and decreases retrograde vesicle transport (30, 31, 41). Thus, our results indicate that JNK phosphorylation activates GSK3β in both direct and indirect manners and can suppress RAT in DPN.

In our DPN model, delay of NCVs was identified as a neuropathic symptom, but thermal hyperalgesia and reduction of IENFD were not. Hyperalgesia generally becomes hypoalgesia during the development of DPN. Because our model corresponds to this transition, no abnormal thermal disturbance was observed. Otherwise, the effects of M1 macrophages on insulin sensitivity may be different between small nonmyelinated fibers and large myelinated fibers. Sugimoto et al. revealed that the insulin receptor is mainly expressed in the axonal membrane and the membranes of the Schwann cells of the paranodal area of myelinated fibers in the SNs (56). These results suggest that M1 macrophage infiltration is likely to elicit greater insulin resistance in myelinated fibers than nonmyelinated fibers. Thus, our proposed pathogenic mechanism may be less involved in the onset and progression of nonmyelinated small fiber neuropathy in early-stage diabetic neuropathy.

RAGE is a multiligand receptor that binds to AGEs, high mobility group box-1 protein (HMGB1) and S100A (7). The contribution of HMGB1 to macrophage polarization may not be greater than that of AGEs in DPN because the stimulation of macrophages with HMGB1 in vitro failed to induce M1 polarization in this study. Regarding the serum level of AGEs, CML was significantly increased, as shown by LC-MS/MS, which is the preferred technique to accurately identify and quantify individual AGEs (34). CML is the most important active center of AGEs and can bind to the V domain of RAGE (57). By binding to RAGE, CML can cause an increase in the production of proinflammatory cytokines such as IL-1β, IL-6, and TNF-α, leading to the occurrence of various diseases (58). These findings suggest that serum CML can play a pivotal role in the development of DPN via the M1 polarization of macrophages in peripheral nerve tissue, particularly in the early stages of DPN.

To date, no radical treatment for DPN has been established. M2 macrophage polarization is thought to be a therapeutic option for various peripheral neuropathies, including DPN (9, 32, 59, 60). In this study, similar to the results of Juranek et al., diabetic BMRN mice showed higher numbers of infiltrating M2 macrophages in the SN than diabetic BMWT mice, which prevented the onset of DPN and attenuated insulin sensitivity (9). These results suggest that modulating RAGE in macrophages is a potential therapeutic strategy, particularly in the early stage of DPN in T1D. On the other hand, macrophages have more detailed categorizations based on disease type (61). Macrophage infiltration in the SN is also thought to be a pathogenic mechanism in the advanced stage of DPN, and the population can change during the progression of DPN (19). Therefore, to establish a treatment for DPN using RAGE in macrophages, a more detailed analysis is required to classify infiltrated macrophages, depending on the stage of DPN in the future.

Our study has some limitations. First, we assessed DPN in STZ-induced T1D mice but not T2D mice. The main pathogenesis of T1D is insulin shortage due to a marked reduction in cells. Because T1D subjects can exhibit insulin resistance with a phenotype that is less consistent with metabolic syndrome, the pathophysiology of insulin resistance may be different from that of T2D (62). However, since the change in macrophage polarization due to the deletion of RAGE could ameliorate insulin resistance with NCVs and RAT in this study, insulin resistance is likely to be involved in the main mechanism downstream of macrophage infiltration in the early stage of T1D DPN. Second, insulin sensitivity in the SN reflects the sensitivity of both neuronal cells and Schwann cells. As mentioned previously, mice with conditional deletion of insulin receptors and IGF-1 receptors specifically in Schwann cells show thinner myelin and delayed NCVs, which are similar to the characteristics of DPN (51). Myelination can increase the amount of retrograde vesicle transport in cocultures of DRG and Schwann cells in vitro (63). These results indicate that insulin resistance in Schwann cells may also be implicated in RAT deficits in DPN. Third, it is difficult to confirm our results in human DRG cells because of the difficulties in obtaining human DRG tissue for primary culture and the lack of a human DRG cell line that is currently available. To overcome this problem, human stem cells such as induced pluripotent stem cells, which can differentiate into DRG neurons, may be used to confirm our results in the future.

In conclusion, the activation of RAGE signaling through chronic hyperglycemia skews the polarity of macrophages toward the proinflammatory M1 phenotype, which induces local inflammation in the SN, disturbing RAT and insulin signaling and resulting in the development of early neuropathy in experimental T1D (Supplemental Figure 9, B and C). These findings suggest a mechanism of the protective effect of RAGE deletion on the onset of T1D DPN, characterizing a crucial role of macrophage-associated inflammation in DPN pathogenesis.

## Methods

### Chemicals, reagents, and antibodies.

Chemicals, reagents, and antibodies used in this study were summarized in Supplemental Tables 2 and 3.

### Animals.

Eight-week-old male C57BL/6 WT and RN were used in the study. RN were generated as described by Myint and colleagues (7). The mice were maintained in a temperature-controlled (24°C) facility with a strict 12-hour light/dark cycle and were fed a regular chow diet (CE-2 rodent diet, CLEA Japan Inc.) ad libitum. Diabetes was induced by a single i.p. injection of STZ (S0130, Merck) in citrate buffer at a dose of 100 mg/kg for 3 consecutive days. Blood glucose levels were measured 7 days after the first STZ injection in samples obtained by tail prick using a OneTouch Verio IQ test (Johnson and Johnson) and a Glutest mint (Sanwa Kagaku Kenkyusho Co.). Animals with blood glucose levels > 16.5 mmol/L (300 mg/dL) were considered diabetic. Animals had blood glucose tests and were weighed monthly. Animals were followed for an additional 8 weeks after diabetes induction. Nondiabetic mice served as controls.

### Measurement of AGEs in mice serum using LC-MS/MS.

The internal standards of [^2^H_2_] CML was purchased from PolyPeptide Laboratories, and [^13^C_6_] lysine was purchased from Cambridge Isotope Laboratories. The AGEs content in each sample was measured by LC-MS/MS using a TSQ Quantiva triple-stage quadrupole mass spectrometer (Thermo Fisher Scientific), as described previously (34). Briefly, 5 μL of serum was reduced with 100 mmol/L sodium borohydride in 50 mmol/L sodium borate buffer (pH 9.1) at 25°C for 4 hours. The internal standards were added to samples, which were then hydrolyzed with 1 mL of 6 mol/L HCl at 100°C for 18 hours. The dried samples were resuspended in 1 mL of distilled water and passed over a Strata-X-C column (Phenomenex), which was prewashed with 1 mL of methanol and equilibrated with 1 mL of distilled water. The column was then washed with 3 mL of 2% formic acid and eluted with 2 mL of 7% ammonia. The pooled elution fractions were dried and resuspended in 1 mL 20% acetonitrile containing 0.1% formic acid. The samples (10 μL) were subjected to LC-MS/MS. LC was conducted on a ZIC-HILIC column (150 × 2.1 mm, 5 μm; Merck Millipore). The mobile phase consisted of solvent A (distilled water containing 0.1% formic acid) and solvent B (acetonitrile containing 0.1% formic acid). The flow rate was 0.2 mL/minutes, and the column was maintained at 40°C. The retention times for AGEs and lysine were approximately 12 and 14 minutes, respectively. AGEs, amino acids, and their internal standards were detected by electrospray ionization and positive ion mass spectrometric multiple reaction monitoring. The parent ions of CML, [^2^H_2_] CML, lysine, and [^13^C_6_] lysine were 205 (*m/z*), 207 (*m/z*), 147 (*m/z*), and 153 (*m/z*), respectively. Fragment ions of 130 (*m/z*) from each parent ion were measured for the analysis of CML and [^2^H_2_] CML in the samples. Lysine and [^13^C_6_] lysine were measured as fragment ions of 84 (*m/z*) and 89 (*m/z*) from parent ion, respectively. The CML was normalized to the lysine content; thus, the data were expressed as mmol/mol lysine (CML).

### BM transplantation.

Ten-week-old male WT mice were lethally irradiated with 9.5 Gy using an x-ray irradiator (MBR-1520R3; Hitachi Medico Co.) with a filter (Cu: 0.5 mm, Al: 2 mm), and the cumulative radiation dose was monitored. The irradiated mice were then immediately administered 2 × 10^6^ BM cells from the femurs of WT or RN through the tail vein. Eight weeks after BM transplantation, diabetes was induced by i.p. injections of STZ. The animals were observed for an additional 8 weeks.

### Insulin treatment.

At starting points and after 8 weeks of diabetes, the mice were anesthetized by isoflurane inhalation and injected with 5 units of insulin (Humulin-R, Lily Japan). The mice were sacrificed 10 minutes after insulin injection, and tissue was harvested, including the SN, DRG, and skin of the hind paw footpad.

### NCVs.

NCVs were examined based on a previous study with a near nerve temperature of 37°C controlled by a heating pad and checked with a thermometer (5, 19). Eight weeks after the onset of diabetes, MNCV and SNCV were evaluated by electric stimulation (MEB-9102, Nihon Kohden Corp.). An average of at least 5 recordings for each mouse was used for the measurements.

### Morphometry of SNs and DRG.

Segments of distal-mid SN were fixed and then embedded in epoxy resin. Sections (1 μm) were stained with toluidine blue and captured under an Axioimager A1 microscope (Carl Zeiss AG). For the morphometric analysis of myelinated fibers, axon area, axon density, axon number, and myelin area were measured using ImageJ ver.1.61 (NIH). The H&E stained 4 μm–thick sections of fixed DRG tissues were captured by Axioimageg A1 (Carl Zeiss AG) (25). The size of ganglion cells containing nuclei was measured at a magnification of ×1,000 by ImageJ version 1.61. The mean values of cell size were obtained from the measurements of all cells on maximum cross-section for each animal. In the same sections, the total number of neuronal cells containing nuclei was counted per ganglion without standardization by DRG area because of the increased density of neuron number due to the miniaturization of whole DRG size in diabetic subjects.

### Tail-flick test.

Eight weeks after the onset of diabetes, the tail-flick response to a thermal stimulus of radiant heat was measured using a heat stimulator (MK-330B, Muromachi Kikai Co.), as described previously (5, 19), because heat perception test using the tail enables to obtain reliable results compared with heat perception tests using plantar paw skin in our facility. Briefly, the animal was gently wrapped in a paper towel and placed on the top of the instrument with the tail in the sensing groove. The tail flick latency was determined by exposing the animal’s tail to a radiant heat source and recording the time taken to remove the tail from the noxious thermal stimulus. The radiation intensity was chosen based on the intensity required to elicit a basal tail flick response of 2–3 seconds in control mice of 13 weeks of age. The temperature for stimulation was set as 56°C. Tail flick latencies were measured 10 times per session at a minimum interval of 10 minutes. Tail movements due to voluntary locomotion were excluded from the measurement.

### Evaluation of IENFD.

IENFD was evaluated using immunofluorescence staining, as previously described (5, 19). The skin of hind foot pads from the plantar surfaces of the left and right hind paws was fixed at 4°C with Zamboni’s Fixative (2% paraformaldehyde and 0.2% picric acid in 0.1 mol/L phosphate buffer) for 6–8 hours, rinsed with 30% sucrose in PBS overnight, cryoembedded in mounting media, and sectioned at a thickness of 30 μm. Epidermal nerve fibers were labeled with an anti–protein gene product 9.5 (anti-PGP9.5) antibody (1:500, Z5116, Agilent), and the dermis was labeled with an anti-CK 5/6 antibody (1:200, M7237, Agilent) and incubated with the following secondary antibodies: Alexa 488–conjugated donkey anti–mouse IgG (1:500, Thermo Fisher Scientific) and Alexa 594–conjugated donkey anti–mouse IgG (1:500, Thermo Fisher Scientific). Approximately 16 sequential images were captured at intervals of 2 μm and flattened in each frame with a CellVoyager CQ1 confocal image cytometer (Yokogawa Electric Corp.). An average of 5 frames were evaluated per section, and 3 sections were measured for each footpad. IENFD data are presented as the mean number of fibers crossing dermal-epidermal junction in the epidermis per linear millimeter of epidermis from a total of 6 sections per animal.

### IHC.

The SN and DRG were fixed immediately upon harvest in 10% neutral-buffered formalin overnight and embedded in paraffin. Then, serial 4 μm–thick deparaffinized sections were immersed in 0.01 mol/L citrate buffer (pH 6.0) and subsequently placed in a pressure chamber (Pascal, DAKO Cytomation) for antigen retrieval at 125°C for 10 minutes. For double immunofluorescence staining, the serial sections of each sample were incubated with the combination of primary antibodies against β3-tubulin (1:200, sc-51670, Santa Cruz Biotechnology) and iNOS (1:100, ab15323, Abcam), β3-tubulin and CD206 (1:500, ab64693, Abcam), or β3-tubulin and CD68 (1:500, ab125212, Abcam) overnight at 4°C. After being washed with TBS-T, the following secondary fluorescent antibodies were added and incubated for 2 hours at room temperature: Alexa 488–conjugated donkey anti–mouse IgG (1:500, A21202, Thermo Fisher Scientific), Alexa 594–conjugated donkey anti–mouse IgG (1:500, A21203, Thermo Fisher Scientific), and Alexa 647–conjugated donkey anti–rabbit IgG (1:500, A31573, Thermo Fisher Scientific). Images were obtained with a CQ1 confocal image cytometer (Yokogawa Electric Corp.).

### Quantitative PCR.

Total RNA was extracted from frozen tissue or cultured cells using TRIzol reagent (Thermo Fisher Scientific) (19). The reverse-transcription reaction was carried out with a Super Script VILO cDNA Synthesis Kit (11754050, Thermo Fisher Scientific). Commercially available primer and probe sets (Gene Expression Assays, Thermo Fisher Scientific) for the target genes *iNos*, *Tnfa*, *Arg1*, *Cd163*, and *Rage* and the internal standard *B2m* were mixed with cDNA and Thunderbird Probe qPCR Mix (QPS-101, Toyobo Inc.). Then, 20 μL of the mixture was loaded onto an optical reaction plate in duplicate. An established reverse transcription PCR (RT-PCR) assay using the relative quantification method (*ΔΔCt*) was conducted in an ABI PRISM 7000 Sequence Detection System (Thermo Fisher Scientific) (19). If mRNA expression of the examined genes was not observed during 1 assay, the thereshold value was regarded as 45 cycles.

### Immunoblotting.

Fresh frozen tissue sections of the SN were homogenized using a homogenizer (Iuchi-AS ONE) in ice-cold lysis buffer composed of 50 mmol/L Tris-HCl (pH 8.0), 1.37 mmol/L NaCl, 1% NP40, and 10% glycerol with a protease inhibitor cocktail (Nichirei Corp.). The lysates were centrifuged at 17,980*g* for 15 minutes, and protein concentrations were measured using Bradford assay reagent (Bio-Rad) with BSA as the standard. Protein (20 μg) was heated at 70°C for 15 minutes. Denaturized protein samples were separated by SDS-PAGE, followed by electrical transfer onto PVDF membranes. After being blocked with 5% BSA in TBS-T, the membrane was incubated with primary antibodies against phospho-JNK (1:4,000, 9251, Cell Signaling Technology), total JNK (1:2,000, sc-474, Santa Cruz Biotechnology), phospho-AKT (1:4,000, 4060, Cell Signaling Technology), total AKT (1:4,000, 4685, Cell Signaling Technology), phospho-GSK3β (1:4,000, 9322, Cell Signaling Technology), total GSK3β (1:1,000, 27C10, Cell Signaling Technology), and β-actin (1:1,000, sc-1615, Santa Cruz Biotechnology) in Can Get Signal Immunoreaction Enhancer Solution (NKB-101T, Toyobo Inc.) overnight at 4°C. Antigen detection was performed using chemiluminescence (ECL Western Blotting Detection Reagents, GE Healthcare Bio-Sciences Corp.) with horseradish peroxidase–conjugated secondary antibodies. Membranes were exposed to Amersham Hyperfilm ECL (GE Healthcare Bio-Sciences Corp). Band density was analyzed by densitometric scanning with ImageJ and normalized to the expression of nonphosphorylated proteins or β-actin.

### Retrograde FG neurotracer labeling.

To label neurons in the DRG with intraplantar injection of a tracer, the mice were anesthetized by isoflurane inhalation. A total volume of 5.0 μL of 5% FG solution (Biotium Inc.) in 0.9% saline was injected into a right-side hindlimb footpad using a 10 μL Hamilton syringe. L4 and L5 DRG were harvested 5 days after FG administration, fixed in 10% neutral-buffered formalin overnight, and embedded in paraffin. Deparaffinized sections (4 μm thick) were immersed in 0.01 mol/L citrate buffer (pH 6.0) and subsequently placed in a pressure chamber (DAKO Cytomation) for antigen retrieval at 125°C for 10 minutes. Sections were incubated with primary antibodies against FG (1:200, AB153-1, Merck) and β3-tubulin (1:200, sc-51670, Santa Cruz Biotechnology) overnight at 4°C. After washing with TBS-T, secondary fluorescent antibodies were added and incubated for 2 hours at room temperature. Images were obtained with a CQ1 confocal image cytometer (Yokogawa Electric Corp.). To quantify retrograde labeling, we counted the number of FG-labeled neurons and calculated the FG^+^ rate (%). Additionally, we measured the mean gray value (MGV) of FG staining as an intensity and β3-tubulin staining in DRG sections and calculated the relative intensity of FG staining.

### RAW264.7 cell activation.

The macrophage cell line RAW264.7 was purchased from RIKEN-BRC (Ibaraki) and cultured in DMEM with 5.6 mmol/L glucose, 10% heat-inactivated FBS (10% FBS), 100 U/mL penicillin, and 100 μg/mL streptomycin (1% PS). RAW264.7 cells were treated and activated with either 200 μg/mL BSA (Vehicle) (Fujifilm Wako Pure Chemical Corp.), 200 μg/mL AGE-BSA (AGE) (2221-10, BioVision Inc.), 1.0 μg/mL HMGB1 (1690-HMB, R&D Systems), or 1.0 μg/ml LPS (2630, Merck) for 12 hours.

### Primary DRG neuronal culture.

Primary cultures of sensory neurons in the DRG of adult mice were prepared as previously described (5). Briefly, DRG from the cervical to the lumbar level were dissected and incubated with 2.0 mg/mL collagenase (CLS-3, Worthington Biochemicals) and 50 U/mL dispase (354235, Corning) and subjected to density gradient centrifugation (5 minutes, room temperature, 200*g*) with 30% Percoll PLUS (28-9038-34 AA, GE Healthcare Bio-Sciences Corp.) to eliminate the myelin sheath. Dissociated DRG neurons were suspended in DMEM: nutrient mixture F-12 (DMEM/F-12) (Fujifilm Wako Pure Chemical Corp.) with 10% FCS and 1% PS and seeded onto 10 μg/mL poly-L-lysine–coated (P1524, Merck) and 10 μg/mL laminin-coated (120–05751, Fujifilm Wako Pure Chemical Corp.) wells of 8-well chamber slides (Nunc Lab-Tek Chamber Slide System, 177402PK, Thermo Fisher Scientific). After being incubated in serum-containing medium for 16 hours, the neurons were maintained for 24 hours (for coculture experiments) or 32 hours (for monoculture experiments) in DMEM/F12 with 2% B-27 (A3653401, Thermo Fisher Scientific).

### DRG neuron-RAW264.7 cell coculture.

DRG neurons from adult mice and RAW264.7 cells were maintained separately as described above. A DRG neuron–RAW264.7 coculture system was prepared by seeding RAW264.7 cells into wells containing DRG neurons after monoculture for 24 hours. Inactivated RAW264.7 cells were suspended in DMEM/F-12 with 10% FCS and 1% PS and added to each well at a density of 1.0 ×10^5^ cells/ mL, and the cells were cultured in medium containing 10% FBS. After 12 hours, the cells were switched to DMEM/F12 with 2% B-27 and 1.0% FCS and treated with either 200 μg/ mL BSA (vehicle; Merck KGaA) or 200 μg/mL AGE-BSA (AGE) (2221-10, BIoVision Inc.) for an additional 12 hours.

### Immunocytochemistry.

DRG neuron monocultures and DRG neuron–RAW264.7 cell cocultures were maintained on chamber slides, fixed with 4% paraformaldehyde for 15 minutes at room temperature, and permeabilized with 0.4% Triton X-100 in PBS for 5 minutes. The cells were incubated with primary antibodies against iNOS (1:100, ab15323, Abcam), phospho-JNK (1:100, 9251, Cell Signaling Technology), phospho-AKT (1:100, 4060, Cell Signaling Technology), phospho-GSK3β (1:4,000, 9322, Cell Signaling Technology), and β3-tubulin (1:200, sc-51670, Santa Cruz Biotechnology) overnight at 4°C. After the samples were washed with 0.4% Triton X-100 in PBS, secondary fluorescent antibodies were added and incubated for 2 hours at room temperature. Images were obtained with a CQ1 confocal image cytometer (Yokogawa Electronic Corp.).

### Fluorescence time-lapse microscopy.

DRG neuron–RAW264.7 cell cocultures and DRG neuron monocultures were exposed to 100 nmol/L LysoTracker Red (L7528, Thermo Fisher Scientific) for 1 hour and switched into fresh media containing Oxyrase (Oxyrase Inc.). Subsequently, in the insulin-minus or insulin-plus experiment shown in Figure 8, A–F, the neurons were maintained in DMEM/F12 without B27 but with 2.5 mg/mL transferrin (201-18081, Fujifilm Wako Pure Chemical Corp.), 50 mmol/L putrescine (100441, MP Biomedicals), 15 μmol/L selenium (196-12622, Fujifilm Wako Pure Chemical Corp.), and 10 μmol/L progesterone (160-24511, Fujifilm Wako Pure Chemical Corp.) in the presence or absence of 1 unit/mL insulin (Humulin-R; Lily Japan). In the experiments with an insulin/IGF-1 receptor antagonist shown in Figure 8, G–L, or with TNF-α with or without a JNK inhibitor shown in Figure 8, M–R, neurons were maintained in DMEM/F12 with 2% B27 and were pretreated with PBS containing 0.1% BSA (vehicle), 300 nmol/L, or 500 nmol/L BMS-754807 (MedChemExpress); 20 ng/mL TNF-α (410-MT, R&D Systems); or 20 ng/mL TNF-α + 50 nmol/L JNK inhibitor (SP600125) (1496, TOCRIS) as indicated in the text. Time-lapse microscopy was performed using a CQ1 confocal image cytometer (Yokogawa Electrical Corp.) in a water-heated built-in microscope stage at 3 °C and 5% CO_2_. Digital images were acquired every 2 seconds for 4 minutes (121 frames).

### Analysis of organelle movement.

Analysis of organelle movement in the axon was performed as described in previous articles (46). Three to 5 experiments were performed per each group. For each experiment, 4–6 fields of neurons were captured randomly. To analyze intraaxonal organelle movement, 100 μm midaxon segments that were clearly linked to a specific neuronal cell body were selected. Kymographs were generated from sequential images from time-lapse capture by drawing regions of interest along the axon in the anterograde direction from the cell body toward the distal axon tip using ImageJ. In total, 17–21 kymographs were generated per each group. Fluorescence puncta were tracked from the beginning to the end of the kymograph to categorize movement as stationary (<5 μm), anterograde (≥5 μm; lines sloped to the right), retrograde (≥5 μm; lines sloped to the left), and bidirectional (zigzagged lines). To analyze motility, all the lines on the kymographs were tracked manually. The run length and the number of stacked time-lapse images, which represents the run time (an image = 2 seconds), were measured. The velocities of every movement were calculated by dividing the run length by the run time. Statistical analysis was performed separately for Figure 8, A–F, and Figure 8, G–R, because of the difference of culture condition. Statistical analysis for Figure 8, G–R, was performed among 5 groups (vehicle, 300 nM BMS-754807, 500 nM BMS-754807, TNF-α, and TNF-α + SP600125), and the results are presented separately in Figure 8, G–L, and Figure 8, M–R, for better visibility. Therefore, same results of Vehicle were used in Figure 8, G–R.

### Statistics.

The data are expressed as the mean ± SD, and individual data points are shown in dot plots. Statistical analysis was carried out using GraphPad Prism 8 software. Where appropriate, the data were subjected to unpaired 2-tailed Student’s *t* test, 1-way ANOVA with post hoc comparisons using Tukey’s post hoc tests, 2-way ANOVA with repeated measures followed by Tukey’s post hoc test, or Pearson’s correlation analysis. Statistical significance is defined as *P* < 0.05.

### Study approval.

All of the procedures followed the *Guide for the Care and Use of Laboratory Animals* (National Academies Press, 2011) and the institutional guidelines of Hirosaki University Animal Experimentation for the care and use of laboratory animals (approval nos. M14005 and M21002).

## Author contributions

S. Osonoi and HM designed the study. S. Osonoi, HM, YT, TS, and KK analyzed the data. S. Osonoi, TS, and S. Ogasawara performed in vivo experiment. S. Osonoi, YT, TS, ST, and KS performed in vitro experiments. S. Osonoi, YT, TS, and KI performed experiments of the BM transplantation model. HS and RN performed experiments of MS for AGEs. S. Osonoi and HM performed the statistical analyses. S. Osonoi, HM, KI, RN, YY, HY, MD, and SY wrote the manuscript.

## Supplementary Material

Supplemental data

Supplemental video 1

Supplemental video 2

Supplemental video 3

Supplemental video 4

Supplemental video 5

Supplemental video 6

Supplemental video 7

Supplemental video 8

Supplemental video 9

Supplemental video 10

## Figures and Tables

**Table 1:**
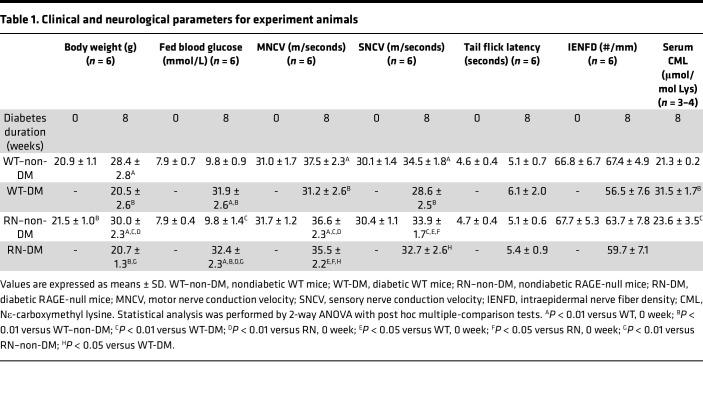
Clinical and neurological parameters for experiment animals

**Table 2:**
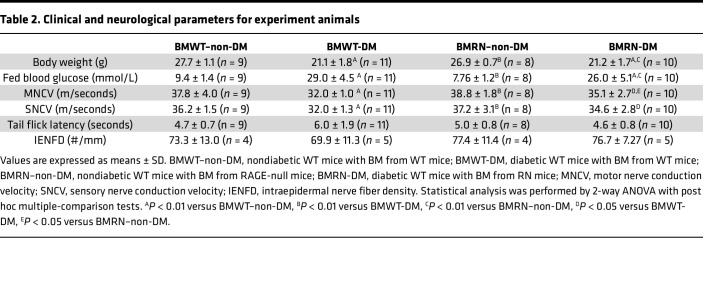
Clinical and neurological parameters for experiment animals

## Version 1 12/08/2022

Electronic publication
