# Supplementary material for: RAGE activation in macrophages and development of experimental diabetic polyneuropathy
Source: JCI Insight. 2022 Dec 8;7(23):e160555. doi: 10.1172/jci.insight.160555 (PMC9746912; doi:10.1172/jci.insight.160555)
Supplement: Supplemental data [file jciinsight-7-160555-s152.pdf]

Supplementary Table 1. Morphometric data on myelinated fibers of sciatic nerves

Group	Fiber area (μm^2)	Axon area (μm^2)	Myelin area (μm^2)	Count number	Count area (μm^2)	Density (count number/mm^2)
0 week WT-non DM (n=5)	11.1 ± 0.9	4.8 ± 0.6	6.3 ± 0.4	553.0 ± 38.5	15293.2 ± 953.6	36326.6 ± 4355.6
8 weeks after diabetic induction						
WT-non DM (n=6)	14.1 ± 1.6*	5.9 ± 0.7*	8.2 ± 0.9*	526.0 ± 54.7	15378.0 ± 1900.7	34335.8 ± 1759.7
WT-DM (n=6)	12.0 ± 1.0†	5.0 ± 0.5†	6.9 ± 0.8‡	507.0 ± 72.5	14119.4 ± 1935.4	36055.8 ± 3743.2
0 week RN-non DM (n=5)	11.2 ± 1.0	4.7 ± 0.5	6.7± 0.5	526.0 ± 54.6	14420.6 ± 660.5	35120.4 ± 3749.2
8 weeks after diabetic induction						
RN-non DM (n=6)	13.9 ± 1.6§	5.8 ± 0.7§	8.1± 1.0§	509.8 ± 83.2	15167.8 ± 2588.0	33739.1 ± 2782.0
RN-DM (n=6)	13.9 ± 1.6§,‖	5.8 ± 0.5§,‖	8.0 ± 0.8§,‖	535.7 ± 49.1	14211.3 ± 2912.2	38530.9 ± 5616.3

Values are expressed as means ± SD. WT-non DM, non-diabetic wild-type mice; WT-DM, diabetic wild-type mice; RN-non DM, non-diabetic RAGE-null mice; RN-DM, diabetic RAGE-null mice; Statistical analysis was performed by one-way ANOVA with Turkey's multiple-comparison test. *P* Values < 0.05 were considered significant; *p<0.01 vs 0W WT-non DM, †p<0.01 vs WT, ‡p<0.05 vs WT, §p<0.01 vs 0W RN-non DM, ‖p<0.05 vs WT-DM.

Supplementary Table 2. List of chemicals and reagents used in this study

Chemical / Reagent name	Catalog No.	Vendor	Experiments	Concentration
AGE-BSA (AGE)	2221-10	BioVision Inc (CA, US))	Cell culture	200 μg/mL
HMBG1	1690-HMB	R&D Systems (MN, US)	Cell culture	1.0 μg/mL
Lipopolysaccharide (LPS)	2630	Merck KGaA (Darmstadt, Germany)	Cell culture	1.0 μg/mL
BMS-754807	HY-10200	MedChemExpress (NJ, US)	Cell culture	300 and 500 nmol/L
TNFα	410-MT	R&D Systems (MN, US)	Cell culture	20 ng/mL
SP600125	1496	TOCRIS (Bristol, UK)	Cell culture	50 nmol/L
Humulin-R (Insulin)	872492	Eli Lilly Japan (Hyogo, JP)	Cell culture	1.0 unit/mL
Transferrin	201-18081	Fujifilm Wako Pure Chemical Corp (Tokyo, JP)	Cell culture	2.5 mg/mL
Puterescine	100441	MP Biomedicals (CA, US)	Cell culture	50 mmol/L
Selenium	196-12622	Fujifilm Wako Pure Chemical Corp (Tokyo, JP)	Cell culture	15 μmol/L
Progesterone	160-24511	Fujifilm Wako Pure Chemical Corp (Tokyo, JP)	Cell culture	10 μmol/L
B-27™	A3653401	Thermo Fisher Scientific (MA, USA)	Cell culture	2%
LysoTracker™ Red	L7528	Thermo Fisher Scientific (MA, USA)	Cell culture	100 nmol/L
Poly-L-lysine	A21202	Thermo Fisher Scientific (MA, USA)	Cell culture	10 μg/mL
Laminin	A21203	Thermo Fisher Scientific (MA, USA)	Cell culture	10 μg/mL
Collagenase	CLS-3	Worthington Biochemicals (NJ, US)	Cell culture	2.0 mg/mL
Dispase	354235	Corning, Corning (NY, US)	Cell culture	50 unit/mL
Percoll PLUS™	28-9038-34 AA	GE Healthcare Bio-Sciences Corp (IL, US)	Cell culture	30%
DMEM High glucose	043-30085	Fujifilm Wako Pure Chemical Corp (Tokyo, JP)	Cell culture	
DMEM/F-12	048-29785	Fujifilm Wako Pure Chemical Corp (Tokyo, JP)	Cell culture	

Supplementary Table 2. List of chemicals and Reagents used in this study (continued)

Chemical / Reagent name	Catalog No.	Vendor	Experiments	Concentration
Bovine serum albumin	017-15141	Fujifilm Wako Pure Chemical Corp (Tokyo, JP)	Cell culture	
Streptozotocin (STZ)	S0130	Merck KGaA (Darmstadt, Germany)	Animal study	100 mg/kg
Hydroxystilbamidine (Fluoro-gold^{TM})	80014	Biotium Inc (CA, US)	Animal study	5%

Supplementary Table 3. List of Antibodies used in this study

Antibody name	Species	Catalog No.	Vendor	Experiments	Dilutions
CD68	Rabbit	ab125212	Abcam plc. (Cambridge, UK)	IF	1:500
iNOS	Rabbit	ab15323	Abcam plc. (Cambridge, UK)	IF, IC	1:100 (IF,IC)
Mannose Receptor (CD206)	Rabbit	ab64693	Abcam plc. (Cambridge, UK)	IF	1:200
Protein gene product 9.5 (PGP 9.5)	Rabbit	Z5116	Agilent (CA, USA)	IF	1:500
Cytokeratin 5/6 (CK 5/6)	Mouse	M7237	Agilent (CA, USA)	IF	1:200
Fluoro-gold	Rabbit	AB153-I	Merck KGaA (Darmstadt, Germany)	IF	1:200
β-tubulin III	Mouse	sc-51670	Santa Cruz biotechnology, Inc. (CA, USA)	IF, IC	1:200 (IF, IC)
Phospho-JNK	Rabbit	#9251	Cell Signaling Technology, Inc., (MA, USA)	WB, IC	1:4000 (WB), 1:100(IC)
Total JNK	Rabbit	sc-474	Santa Cruz Biotechnology, Inc., (TX, USA)	WB	1:2000
Phospho-AKT	Rabbit	#4060	Cell Signaling Technology, Inc.(MA, USA)	WB, IC	1:4000 (WB), 1:100 (IC)
Total AKT	Rabbit	#4685	Cell Signaling Technology, Inc.(MA, USA)	WB	1:4000
Phospho-GSK3β	Rabbit	#9322	Cell Signaling Technology, Inc.(MA, USA)	WB, IC	1:4000 (WB), 1:100 (IC)
Total GSK3β	Rabbit	#27C10	Cell Signaling Technology, Inc.(MA, USA)	WB	1:1000
β-actin	Rabbit	sc-1615	Santa Cruz Biotechnology, Inc.(MA, USA)	WB	1:1000
Alexa fluore 488 anti-mouse IgG (H+L)	Donkey	A21202	Thermo Fisher Scientific (MA, USA)	IF	1:500
Alexa fluore 594 anti-mouse IgG (H+L)	Donkey	A21203	Thermo Fisher Scientific (MA, USA)	IF	1:500
Alexa fluore 647 anti-rabbit IgG (H+L)	Donkey	A31573	Thermo Fisher Scientific (MA, USA)	IF	1:500

IF; immunofluorescence, IC; immunocytochemistry, WB; Western blotting.

Supplementary Figure 1

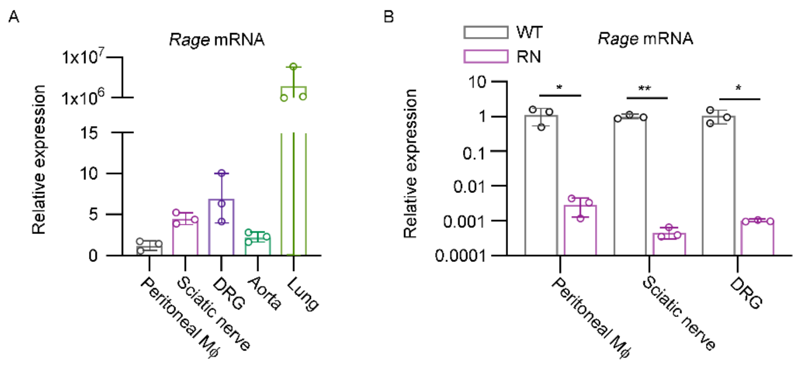

Supplementary Figure 1: mRNA expression of RAGE in various organs.

(A) Relative mRNA expression of *Rage* in peritoneal macrophages, sciatic nerve, DRG, lung and aorta from wild-type mice (WT). (B) Relative mRNA expression of *Rage* in each organ from WT compared to RAGE-null mice (RN). Data are presented as the mean ± SD; n = 3 mice / group. Statistical analysis was performed by Student's unpaired t test. *P* Values < 0.05 were considered significant; * *P* < 0.05, ** *P* < 0.01.

Supplementary Figure 2

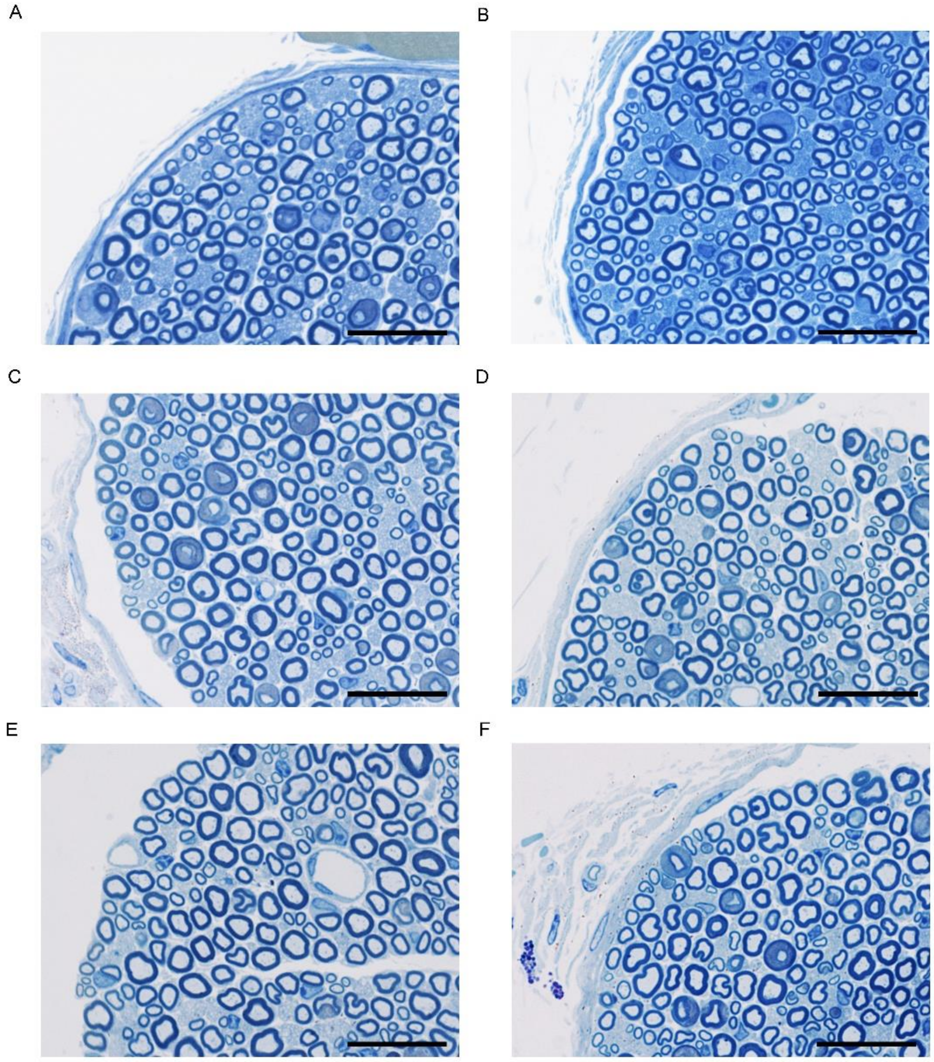

Supplementary Figure 2, related to Supplementary Table 3: Pathological evaluation of sciatic nerves.

Representative images of the sciatic nerves embedded in epoxy resin and stained with toluidine blue for 8 weeks of age non-diabetic wild-type mice (WT–non-DM) (A), non-diabetic RAGE null mice (RN–non-DM) (B), 16 weeks of age WT–non-DM (C), diabetic wild type mice (D), RN–non-DM (E) and diabetic RAGE null mice (F). scale bar = 50 μm.

Supplementary Figure 3

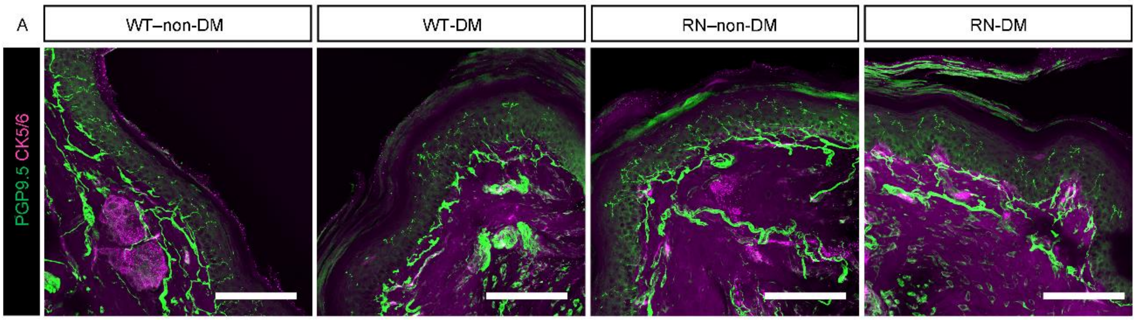

Supplementary Figure 3, related to Table 1: Intra epidermal nerve fibers of mice foot-pad skin.

(A) Representative images of the skin showing intra-epidermal nerve fibers immunostained for PGP9.5 and Cytokeratin 5/6 (CK5/6) (scale bar = 100 μm). Non-diabetic wild-type mice (WT–non-DM), diabetic wild type mice (WT-DM), non-diabetic RAGE null mice (RN–non-DM) and diabetic RAGE null mice (RN-DM).

Supplementary Figure 4

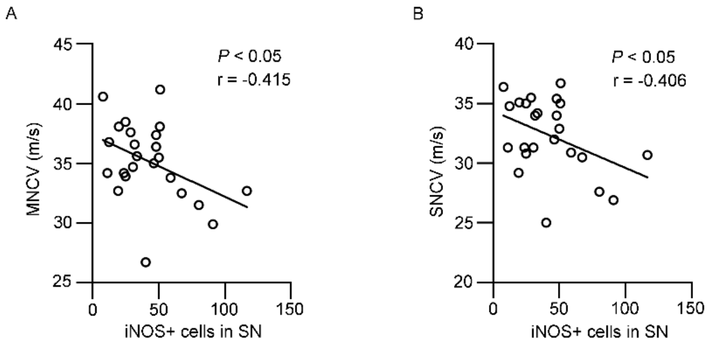

Supplementary Figure 4, related to Table 1 and Figure 1: The correlation between NCVs and iNOS positive macrophages in the sciatic nerves.

(**A**) The correlation between motor nerve conduction velocities (MNCV) or sensory nerve conduction velocities (SNCV) and iNOS positive macrophages in the sciatic nerves (SN) of the experimental mice. Statistical analysis was performed by Pearson's correlation analysis for C. *P* Values < 0.05 were considered significant. NCVs, nerve conduction velocities.

Supplementary Figure 5

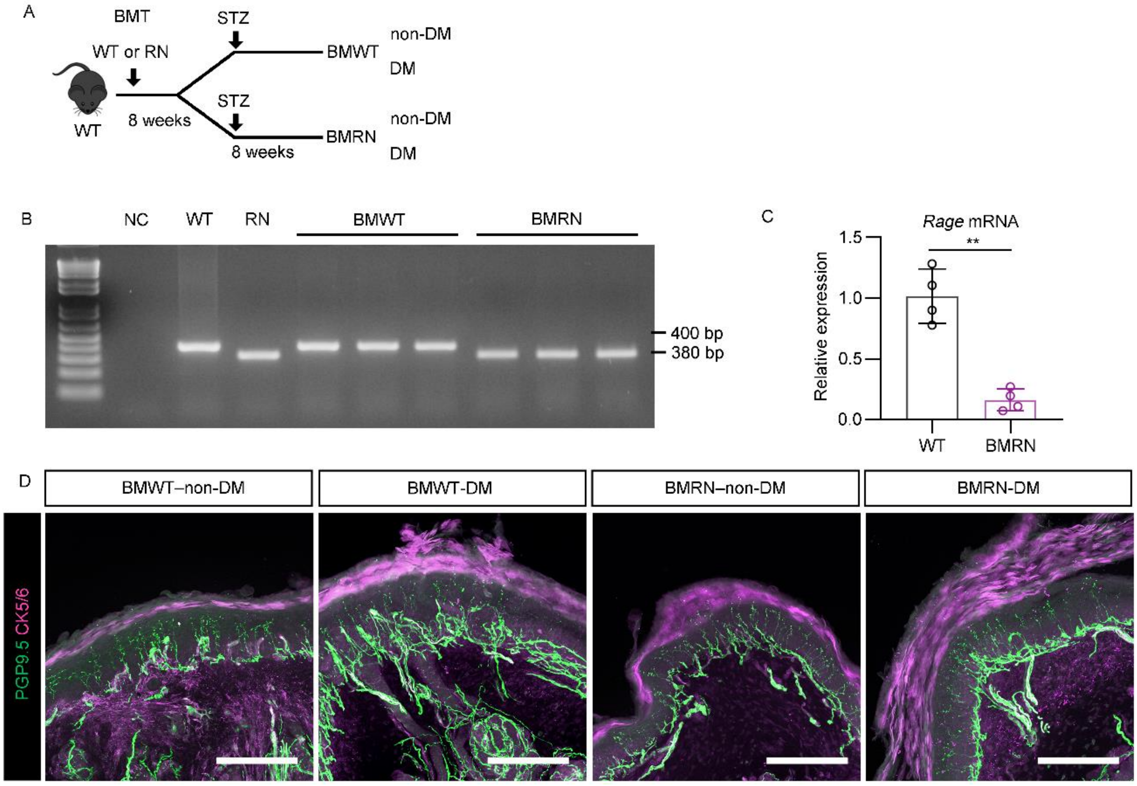

Supplementary Figure 5, related to Table 2: The method of bone marrow transplantation and the evaluation of the genotype of mice and intra epidermal nerve fibers of mice foot-pad skin.

(A) Flowchart of the bone marrow transplantation (BMT) and the induction of diabetes. Non-diabetic wild-type mice with BM from WT mice (BMWT–non-DM), diabetic wild type mice with BM from WT mice (BMWT-DM), non-diabetic wild-type mice with BM from RAGE null mice (BMRN–non-DM) and diabetic wild-type mice with BM from RAGE null mice (BMRN-DM). (B) Genotyping PCR of genome DNA extracted from mice whole blood. Negative control (NC). (C) Relative mRNA expressions of RAGE in the bone marrow from WT and BMRN. (D) Representative images of the skin showing intra-epidermal nerve fibers immunostained for PGP9.5 (scale bar = 100 μm) and Cytokeratin 5/6 (CK5/6). Data are presented as the mean ± SD; n= 4 mice / group for c. Statistical analysis were performed by Student's unpaired t test for c. P Values < 0.05 were considered significant; * $P < 0.05$, ** $P < 0.01$.

Supplementary Figure 6

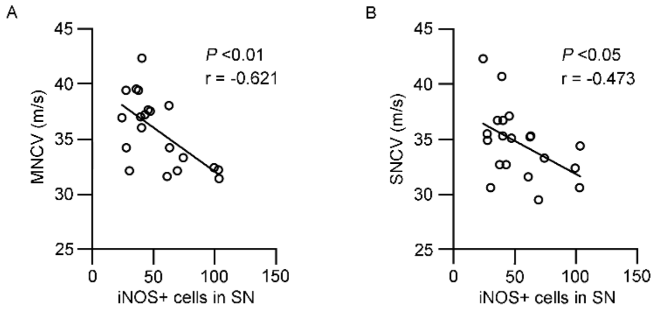

Supplementary Figure 6, related to Table 2 and Figure 4: The correlation between NCVs and iNOS positive macrophages in the SN of bone marrow transplanted mice.

(A) The correlation between motor nerve conduction velocities (MNCV) or sensory nerve conduction velocities (SNCV) (B) and iNOS positive macrophages in the sciatic nerves (SN) of bone marrow transplanted mice. Statistical analysis was performed by Pearson's correlation analysis for C. *P* Values < 0.05 were considered significant. NCVs, nerve conduction velocities.

Supplementary Figure 7

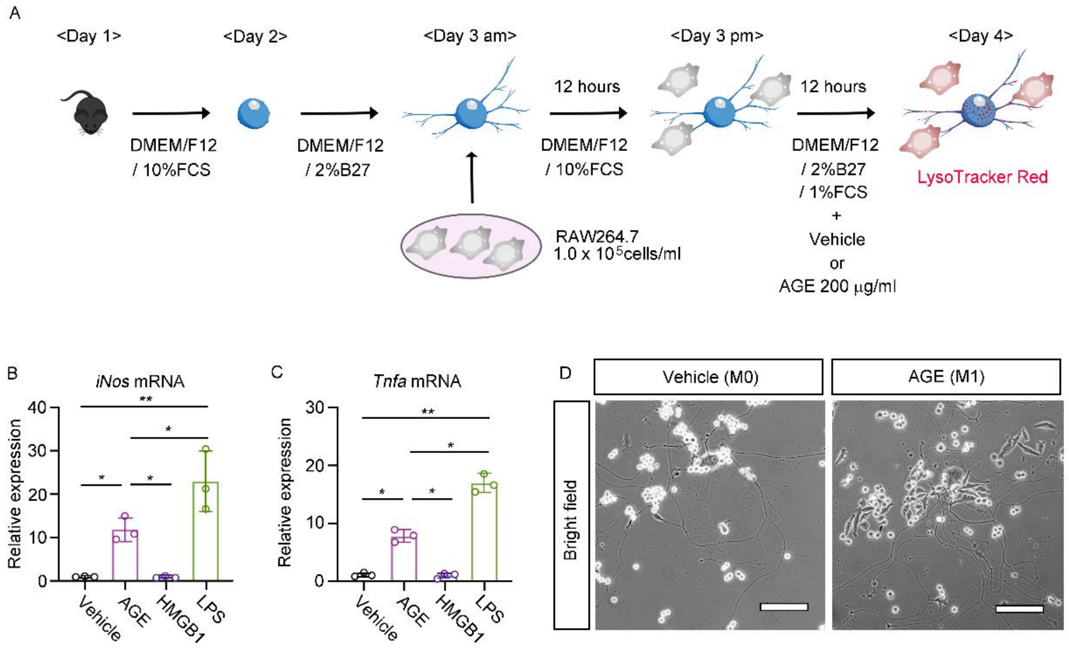

Supplementary Figure 7: AGE stimulation polarizes RAW264.7 macrophages into the inflammatory phenotype.

(A) The methodological procedure of coculture system of the DRG neuron and RAW264.7 cells was shown. (B-C) Relative mRNA expression of *iNOS* (B) and *Tnfa* (C) in RAW264.7 cells stimulated with 200 μg /mL BSA (vehicle), 200 μg /mL AGE, 1.0 μg /mL high mobility group box-1 (HMGB1) or 1.0 μg/mL LPS. (D) Bright field images of direct coculture of DRG neuron and RAW264.7 cells, which were incubated with 200 μg/mL BSA (vehicle) (M0) or 200 μg /mL AGE (M1) for 12 hours (scale bar: 100 μgm). Stimulation with AGE changed the morphology of RAW264.7 into spindle or amoeboid shapes compared to vehicle. Data are presented as the mean ± SD; n= 3 / group. Statistical analysis was performed by one-way ANOVA with Turkey's multiple-comparison test. *P* Values < 0.05 were considered significant; * $P < 0.05$, ** $P < 0.01$.

Supplementary Figure 8

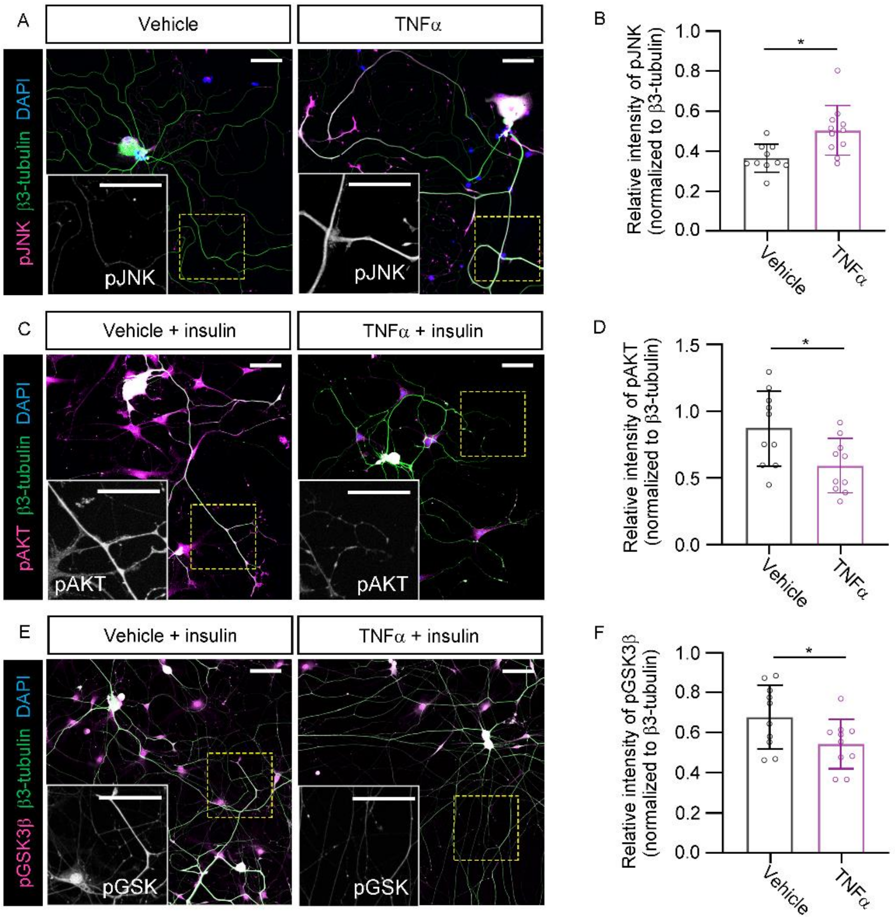

Supplementary Figure 8, related to Figure 7 and 8: in vitro assessment of DRG neurons co-cultured with RAW264.7 macrophages or stimulated with TNFα.

Immunofluorescent images showing phosphorylation of JNK (A), AKT (C) and GSK3β (E) in DRG neurons treated with PBS containing 0.1% BSA (Vehicle) or 20 ng/mL TNFα for 20 minutes (A) and subsequently treated with 1.0 U/mL insulin for 10 minutes (C and E). Neurons were counterstained for β3-tubulin (scale bar: 50 μm). Relative fluorointensity of phosho-JNK (B), phosho-AKT (D) and phosho-GSK3β (F) normalized to β3-tubulin in axons. Data are presented as the mean ± SD, n = 10 fields (x 20) /group from 3 independent experiments. Statistical analysis was performed by Student's unpaired t test. *P* Values < 0.05 were considered significant; * *P* < 0.05.

Supplementary Figure 9

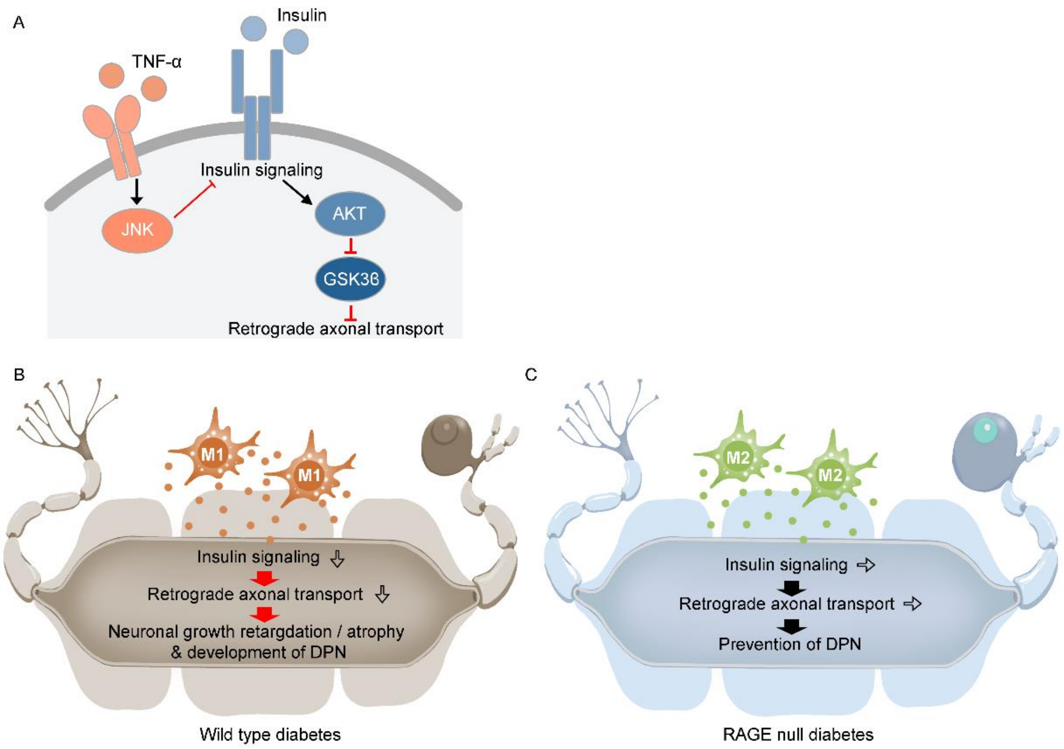

Supplementary Figure 9: A proposed model for the role of RAGE in early stage of DPN in type 1 diabetes.

(A) Associations between Insulin signaling and retrograde axonal transport in the axon of DRG neurons. (B) Hyperglycemia and AGE formation triggers M1 macrophage-associated local inflammation in the sciatic nerves (SN), which induces resistance of the insulin signaling and the decrease of retrograde axonal transport, resulting in neuronal growth retardation/ atrophy and development of DPN of type 1 diabetes. (C) Deletion of RAGE in macrophages skews the polarity towards M2 macrophage phenotype in diabetic condition. Anti-inflammatory environment ameliorates abnormal changes in the SN and prevents the onset of DPN.

Supplementary Video 1: Transport in DRG neuron monocultures after AGE stimulation.

DRG neurons were cultured in chamber slides for 2 days. Axons were pretreated with 200 μg/mL AGE for 12 hours and LysoTracker Red for 1 hour. Time-lapse images of neurons were captured every 2 seconds for 4 minutes. The video frame rate is 10 frames per second.

Supplementary Video 2: Transport in DRG neuron-RAW264.7 cell cocultures after vesicle stimulation.

DRG neurons were monocultured in chamber slides for 24 hours. Subsequently, RAW264.7 cells were seeded onto DRG neurons at 1.0 $\times 10^5$ cells/mL. The cells were cocultured for 12 hours. Axons were pretreated with 200 μg/mL BSA for 12 hours and LysoTracker Red for 1 hour. Time-lapse images of neurons were captured every 2 seconds for 4 minutes. The video frame rate is 10 frames per second.

Supplementary Video 3: Transport in DRG neuron-RAW264.7 cell cocultures after AGE stimulation.

DRG neurons were monocultured in chamber slides for 24 hours. Subsequently, RAW264.7 cells were seeded onto DRG neurons at 1.0 $\times 10^5$ cells/mL. The cells were cocultured for 12 hours. Axons were pretreated with 200 μg/mL AGE for 12 hours and

LysoTracker Red for 1 hour. Time-lapse images of neurons were captured every 2 seconds for 4 minutes. The video frame rate is 10 frames per second.

Supplementary Video 4: Transport in DRG neuron monocultures after insulin withdrawal.

DRG neurons were cultured in chamber slides for 2 days. Axons were pretreated with 2.5 mg/mL Transferrin, 50 mmol/L Putrescine, 15 μmol/L Selenium, 10 μmol/L Progesterone without B27^{TM} supplementation, and LysoTracker Red for 1 hour. Time-lapse images of neurons were captured every 2 seconds for 4 minutes. The video frame rate is 10 frames per second.

Supplementary Video 5: **Transport in DRG neuron monocultures after insulin stimulation.**

DRG neurons were cultured in chamber slides for 2 days. Axons were pretreated with 2.5 mg/mL Transferrin, 50 mmol/L Putrescine, 15 μmol/L Selenium, 10 μmol/L Progesterone, 1.0 U/mL Humulin-R without B27^{TM} supplementation, and LysoTracker Red for 1 hour. Time-lapse images of neurons were captured every 2 seconds for 4 minutes. The video frame rate is 10 frames per second.

Supplementary Video 6: Transport in DRG neuron monocultures under baseline conditions.

DRG neurons were cultured in chamber slides for 2 days. Axons were pretreated with LysoTracker Red for 1 hour and PBS containing 0.1% BSA (Vehicle) for 20 minutes. Time-lapse images of neurons were captured every 2 seconds for 4 minutes. The video frame rate is 10 frames per second.

Supplementary Video 7: Transport in DRG neuron monocultures upon TNFα stimulation.

DRG neurons were cultured in chamber slides for 2 days. Axons were pretreated with LysoTracker Red for 1 hour and 20 ng/mL TNFα for 20 minutes. Time-lapse images of neurons were captured every 2 seconds for 4 minutes. The video frame rate is 10 frames per second.

Supplementary Video 8: Transport in DRG neuron monocultures upon TNFα stimulation in the presence of SP600125.

DRG neurons were cultured in chamber slides for 2 days. Axons were pretreated with 50 nmol/L SP600125 (JNK inhibitor) and LysoTracker Red for 1 hour and 20 ng/mL TNFα with 50 nmol/L SP600125 for 20 minutes. Time-lapse images of neurons were captured

every 2 seconds for 4 minutes. The video frame rate is 10 frames per second.

Supplementary Video 9: Transport in DRG neuron monocultures upon stimulation with 300 nmol/L BMS-754807.

DRG neurons were cultured in chamber slides for 2 days. Axons were pretreated with 300 nmol/L BMS-754807 (insulin receptor/IGF-1 receptor antagonist) and LysoTracker Red for 1 hour. Time-lapse images of neurons were captured every 2 s for 4 minutes. The video frame rate is 10 frames per second.

Supplementary Video 10: Transport in DRG neuron monocultures upon 500 nmol/L BMS-754807 stimulation.

DRG neurons were cultured in chamber slides for 2 days. Axons were pretreated with 500 nmol/L BMS-754807 (insulin receptor/IGF-1 receptor antagonist) and LysoTracker Red for 1 hour. Time-lapse images of neurons were captured every 2 s for 4 minutes. The video frame rate is 10 frames per second.
